# Prohibitin depletion extends lifespan of a TORC2/SGK‐1 mutant through autophagy and the mitochondrial UPR

**DOI:** 10.1111/acel.13359

**Published:** 2021-05-03

**Authors:** Patricia de la Cruz‐Ruiz, Blanca Hernando‐Rodríguez, Mercedes M. Pérez‐Jiménez, María Jesús Rodríguez‐Palero, Manuel D. Martínez‐Bueno, Antoni Pla, Roxani Gatsi, Marta Artal‐Sanz

**Affiliations:** ^1^ Andalusian Centre for Developmental Biology Consejo Superior de Investigaciones Científicas/Junta de Andalucía/Universidad Pablo de Olavide Seville Spain; ^2^ Department of Molecular Biology and Biochemical Engineering Universidad Pablo de Olavide Seville Spain

**Keywords:** autophagy, lipogenesis, mitochondria, prohibitin, SGK‐1, UPR^mt^

## Abstract

Mitochondrial prohibitins (PHB) are highly conserved proteins with a peculiar effect on lifespan. While PHB depletion shortens lifespan of wild‐type animals, it enhances longevity of a plethora of metabolically compromised mutants, including target of rapamycin complex 2 (TORC2) mutants *sgk*‐*1* and *rict*‐*1*. Here, we show that *sgk*‐*1* mutants have impaired mitochondrial homeostasis, lipogenesis and yolk formation, plausibly due to alterations in membrane lipid and sterol homeostasis. Remarkably, all these features are suppressed by PHB depletion. Our analysis shows the requirement of SRBP1/SBP‐1 for the lifespan extension of *sgk*‐*1* mutants and the further extension conferred by PHB depletion. Moreover, although the mitochondrial unfolded protein response (UPR^mt^) and autophagy are induced in *sgk*‐*1* mutants and upon PHB depletion, they are dispensable for lifespan. However, the enhanced longevity caused by PHB depletion in *sgk*‐*1* mutants requires both, the UPR^mt^ and autophagy, but not mitophagy. We hypothesize that UPR^mt^ induction upon PHB depletion extends lifespan of *sgk*‐*1* mutants through autophagy and probably modulation of lipid metabolism.

## Graphical abstract

TORC2/SGK‐1 mutants have impaired mitochondrial homeostasis, lipogenesis and yolk formation plausibly due to alterations in membrane lipid homeostasis. Depletion of the mitochondrial prohibitin complex suppresses sgk‐1 mutant defects, extending lifespan through autophagy and the mitochondrial UPR.

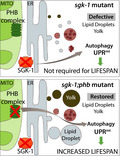

## 1 INTRODUCTION

Mitochondrial function, nutrient signalling and autophagy regulate ageing across phyla. However, their exact mechanisms and interactions in lifespan modulation still remain elusive. The mitochondrial prohibitin (PHB) complex is a strongly evolutionarily conserved ring‐like macromolecular structure (Artal‐Sanz & Tavernarakis, 2009a), important for mitochondrial morphogenesis and membrane maintenance with a poorly understood biochemical function (Hernando‐Rodriguez & Artal‐Sanz, 2018). PHB depletion severely perturbs mitochondrial homeostasis causing an induction of the mitochondrial unfolded protein response, UPR^mt^ (Hernando‐Rodriguez et al., 2018). Intriguingly, PHB depletion has opposing effects on lifespan depending on the genetic background. Loss of PHB shortens lifespan in wild‐type worms, whereas it increases lifespan in different metabolically compromised backgrounds (Artal‐Sanz & Tavernarakis, 2009b). In particular, PHB depletion increases lifespan of the long‐lived Insulin/IGF‐1 receptor mutant *daf*‐*2*(*e1370*), where the induction of the UPR^mt^ is reduced (Gatsi et al., 2014). Analysis of known kinases acting downstream of the insulin receptor revealed that loss of function of SGK‐1 in PHB‐depleted animals results in enhanced longevity and reduced UPR^mt^ activation similar to that observed in PHB‐depleted *daf*‐*2* mutants (Gatsi et al., 2014).

SGK‐1 belongs to the AGC kinase family and is the sole *C. elegans* homologue of the mammalian Serum‐ and Glucocorticoid‐inducible Kinase. In addition to acting in the insulin pathway, SGK‐1 regulates ageing and mitochondrial homeostasis through a parallel pathway, as part of TORC2 (Target Of Rapamycin Complex 2), downstream of RICT‐1. In worms, SGK‐1 regulates development, fat metabolism and lifespan in a complex and controversial manner, partially explained by the different growing conditions, including food source, temperature and presence/absence of FUdR (5‐Fluoro‐2´‐Deoxyuridine; a pyrimidine analogue that inhibits DNA synthesis) to inhibit progeny production (Chen et al., 2013; Evans et al., 2008; Gatsi et al., 2014; Hertweck et al., 2004; Mizunuma et al., 2014; Rahman et al., 2010; Soukas et al., 2009). In this study, we used the *sgk*‐*1* null allele *ok538* (Hertweck et al., 2004), which is consistently long‐lived independently of the bacterial food source, when grown at the standard temperature of 20°C and in the absence of FUdR (Evans et al., 2008; Gatsi et al., 2014; Rahman et al., 2010).

To better understand how SGK‐1 regulates lifespan and mitochondrial function and how PHB deficiency further extends *sgk*‐*1* mutant lifespan, we explored the interaction between PHB and SGK‐1. We show that long‐lived *sgk*‐*1* mutants have altered mitochondrial structure and function, phenotypes that are suppressed by PHB depletion. A transcription factor RNAi screen identified membrane lipid homeostasis as a mechanism implicated in SGK‐1‐mediated maintenance of mitochondrial function. *sgk*‐*1* mutants show altered ER‐mitochondrial contacts, as well as defective lipogenesis and lipoprotein production. Remarkably, all these phenotypes are suppressed by PHB depletion. Furthermore, lifespan analyses showed that autophagy and the UPR^mt^ are required for the enhanced longevity of *sgk*‐*1* mutants upon PHB depletion, while mitophagy is not. Our data suggest that UPR^mt^ induction upon PHB depletion extends lifespan through autophagy and probably modulation of lipid metabolism.

## 2 RESULTS

### 2.1 Prohibitin depletion suppresses the altered mitochondrial structure and function of *sgk*‐*1* mutants

We previously showed that worms lacking the TORC2 component RICT‐1 or the downstream kinase SGK‐1 have an induced mitochondrial unfolded protein response (UPR^mt^) and reduced mitochondrial prohibitin (PHB) levels, showing an unprecedented role for TORC2/SGK‐1 in the regulation of mitochondrial homeostasis (Gatsi et al., 2014). In addition, deletion of *sgk*‐*1* reverts the ageing phenotype of PHB depletion. While depletion of PHB shortens lifespan in wild‐type animals, it further increases that of *sgk*‐*1* mutants (Gatsi et al., 2014). More recent data have confirmed the relevance of mTORC2/SGK‐1 in maintaining mitochondrial permeability (Zhou et al., 2019) and low mitochondrial‐derived ROS levels (Aspernig et al., 2019).

**FIGURE 1:**
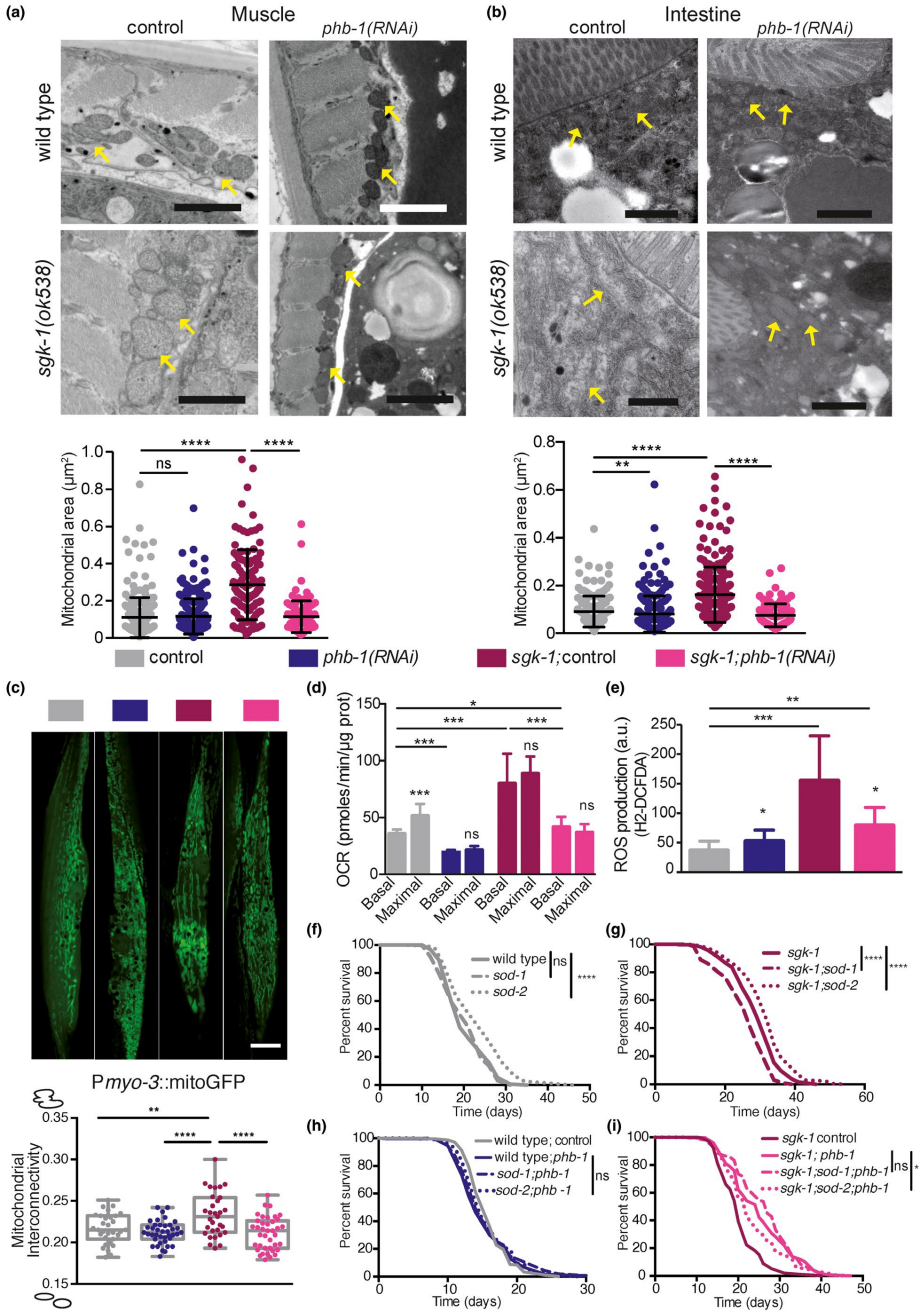
Prohibitin depletion suppresses the altered mitochondrial structure and function of *sgk*‐*1* mutants. Wild‐type animals, *phb*‐*1(RNAi)* treated worms, *sgk*‐*1* mutants and *sgk*‐*1*;*phb*‐*1* depleted animals were analysed (a, b) Electron microscopy images and quantification of mitochondrial area in muscle (a) and intestine (b) at day five of adulthood. Yellow arrows show mitochondria. Mean ± *SD*; ****p < 0.0001, ***p < 0.001, **p < 0.05, *p < 0.5, ns not significant; *t* test, *n* > 100 mitochondria per condition. Bar size: 1 μm. (c) Muscle mitochondria (visualized with P*myo*‐*3*::mitoGFP) at day one of adulthood. Scale bar: 10 μm. Quantitation of mitochondrial interconnectivity. Median ± *SD* of three independent replicates (more than 30 worms and more than 1400 mitochondria per condition ****p ≤ 0.0001, **p ≤ 0.01; Tukey's multiple comparisons test. (d) Seahorse measurements of basal and maximal respiratory capacity at the young adult stage (e) Reactive oxygen species (ROS) levels measured at young adult stage. Mean ± *SD*; ***p < 0.001, **p < 0.05, *p < 0.5, ns not significant; *t* *test*. Combination of three independent replicates. (f–i) Role of ROS in lifespan (f) Lifespan of wild type, *sod*‐*1* and *sod*‐*2* mutants. (g) Lifespan of *sgk*‐*1* mutants, *sgk*‐*1*;*sod*‐*1* and *sgk*‐*1*;*sod*‐*2* double mutants. (h) Lifespan of wild‐type animals, *sod*‐*1* and *sod*‐*2* mutants upon PHB depletion. (i) Effect of PHB depletion in the lifespan of *sgk*‐*1* mutants, *sgk*‐*1*;*sod*‐*1* and *sgk*‐*1*;*sod*‐*2* double mutants. Lifespan replicates and statistics are shown in Table S1

To better define the mitochondrial defect of *sgk*‐*1* deletion mutants and the interaction with PHB, we performed transmission electron microscopy (TEM) analysis. Mitochondria in *sgk*‐*1* mutants were swollen and bigger compared with wild‐type worms in muscle and intestinal cells at day five of adulthood (Figure 1a,b, respectively). This phenotype was also observed at day one of adulthood (Figure S1a,b). Interestingly, while mitochondria became smaller during ageing in wild‐type animals, in *sgk*‐*1* mutants they remained constant, both, in muscle and in intestinal cells (Figure S1b). PHB depletion in otherwise wild‐type worms reduced the mitochondrial size in intestinal but not in muscle cells. Interestingly, PHB depletion drastically reduced the mitochondrial size of *sgk*‐*1* mutants in both tissues (Figure 1a,b). This reduction was not an indirect effect of reduced worm size, since PHB depletion, at day one of adulthood, increased *sgk*‐*1* mutants size (Figure S1c) and, at day five of adulthood, did not alter the TEM worm‐section area (Figure S1d). Supporting the TEM observations, analysis of mitochondrial ultrastructure in muscle cells revealed hyperfused and longer mitochondria in *sgk*‐*1* mutants. The increased mitochondrial interconnectivity of *sgk*‐*1* mutants was suppressed by PHB depletion (Figure 1c).

To unravel if altered mitochondrial size relates to mitochondrial function, we analysed mitochondrial performance in *sgk*‐*1* mutants in the presence or absence of the PHB complex, during ageing. At the young adult stage, *sgk*‐*1* mutants showed a dramatic increase in both, basal and maximal oxygen consumption rate (OCR) compared with wild‐type worms (Figure 1d). The increased respiration rate of *sgk*‐*1* mutants was suppressed by PHB depletion, which also reduced the OCR of wild‐type worms (Figure1c, d). Upon mitochondrial uncoupling, *sgk*‐*1* mutants and *phb*‐*1* depleted worms showed an insignificant spare respiratory capacity, indicating that mitochondria work at their maximal capability (Figure 1d). Furthermore, *sgk*‐*1* mutants showed increased ATP‐linked OCR (Figure S1e) which was suppressed by *phb*‐*1(RNAi*) (Figure S1e), suggesting that oxidative phosphorylation is compromised upon depletion of the PHB complex. Interestingly, *sgk*‐*1* mutants showed increased non‐mitochondrial respiration, which was suppressed by PHB depletion. Lack of PHB also reduced non‐mitochondrial respiration in wild‐type worms (Figure S1f). During ageing, at day six of adulthood, OCR diminished compared with young animals under all conditions except for *phb*‐*1(RNAi)* worms (Figure S1g). Interestingly, PHB depletion increased the OCR of wild‐type and *sgk*‐*1* mutants at day six (Figure S1g). In *sgk*‐*1* mutants, respiration dramatically dropped during ageing, to the level of wild‐type animals.

Increased mitochondrial and non‐mitochondrial respiration could be related to increased reactive oxygen species (ROS) levels. Therefore, we evaluated the levels of ROS in *sgk*‐*1* mutants in the presence and absence of the PHB complex using H2‐DCFDA, a reagent that measures mostly cytosolic ROS. *sgk*‐*1* mutants showed dramatically elevated ROS levels compared with wild‐type worms. In agreement, *sgk*‐*1* mutants also have increased mitochondrial ROS (Aspernig et al., 2019). Depletion of *phb*‐*1* reduced ROS levels in *sgk*‐*1* mutants while mildly increased ROS levels in wild‐type worms (Figure 1e; Artal‐Sanz & Tavernarakis, 2009b). Mitochondrial ROS has been shown to extend lifespan of mutants with high cytoplasmic ROS (Schaar et al., 2015), thus, we checked if mitochondrial ROS contributes to the enhanced lifespan of *sgk*‐*1* mutants upon *phb*‐*1* depletion. Interestingly, treatment with the antioxidant N‐acetyl‐cysteine (NAC) suppressed the longevity conferred by PHB depletion to *sgk*‐*1* mutants without affecting *sgk*‐*1* lifespan (Figure S1i; Table S1). Although at the concentration used NAC also shortened the lifespan of wild‐type and *phb*‐*1* depleted animals (Figure S1h; Table S1), the shortening was more pronounced in the double mutants (10.5%, 11.7% and 16.6%, respectively). We then genetically manipulated cytosolic and mitochondrial ROS levels. Lack of cytosolic superoxide dismutase, *sod*‐*1*, reduced the lifespan of *sgk*‐*1* mutants without affecting wild‐type worms or PHB‐depleted animals (Figure 1f–h; Table S1), thus, increased cytosolic ROS is detrimental for *sgk*‐*1* lifespan. However, PHB depletion protected *sgk*‐*1* mutants from the deleterious effect of cytosolic ROS (Figure 1i; Table S1). Lack of the mitochondrial superoxide dismutase, *sod*‐*2*, increased the lifespan of wild‐type animals, as previously described (Van Raamsdonk & Hekimi, 2009), as well as of *sgk*‐*1* mutants (Figure 1f,g; Table S1), suggesting that mitochondrial ROS is beneficial for *sgk*‐*1* mutants´ lifespan. PHB‐depleted animals were not affected by the lack of *sod*‐*2* (Figure 1h; Table S1). In contrast, *sod*‐*2* was partially required for the lifespan extension conferred by PHB depletion to *sgk*‐1 mutants (Figure 1i; Table S1), suggesting that too much mitochondrial ROS becomes detrimental. Although PHB depletion extends lifespan independently of ROS (Figure S1i; Artal‐Sanz & Tavernarakis, 2009b), these data suggest that mitohormesis is partially involved in the lifespan increase conferred to *sgk*‐*1* mutants.

### 2.2 Altered sterol and lipid homeostasis mimic and exacerbate the UPR^mt^ induction of TORC2/SGK‐1 mutants

**FIGURE 2:**
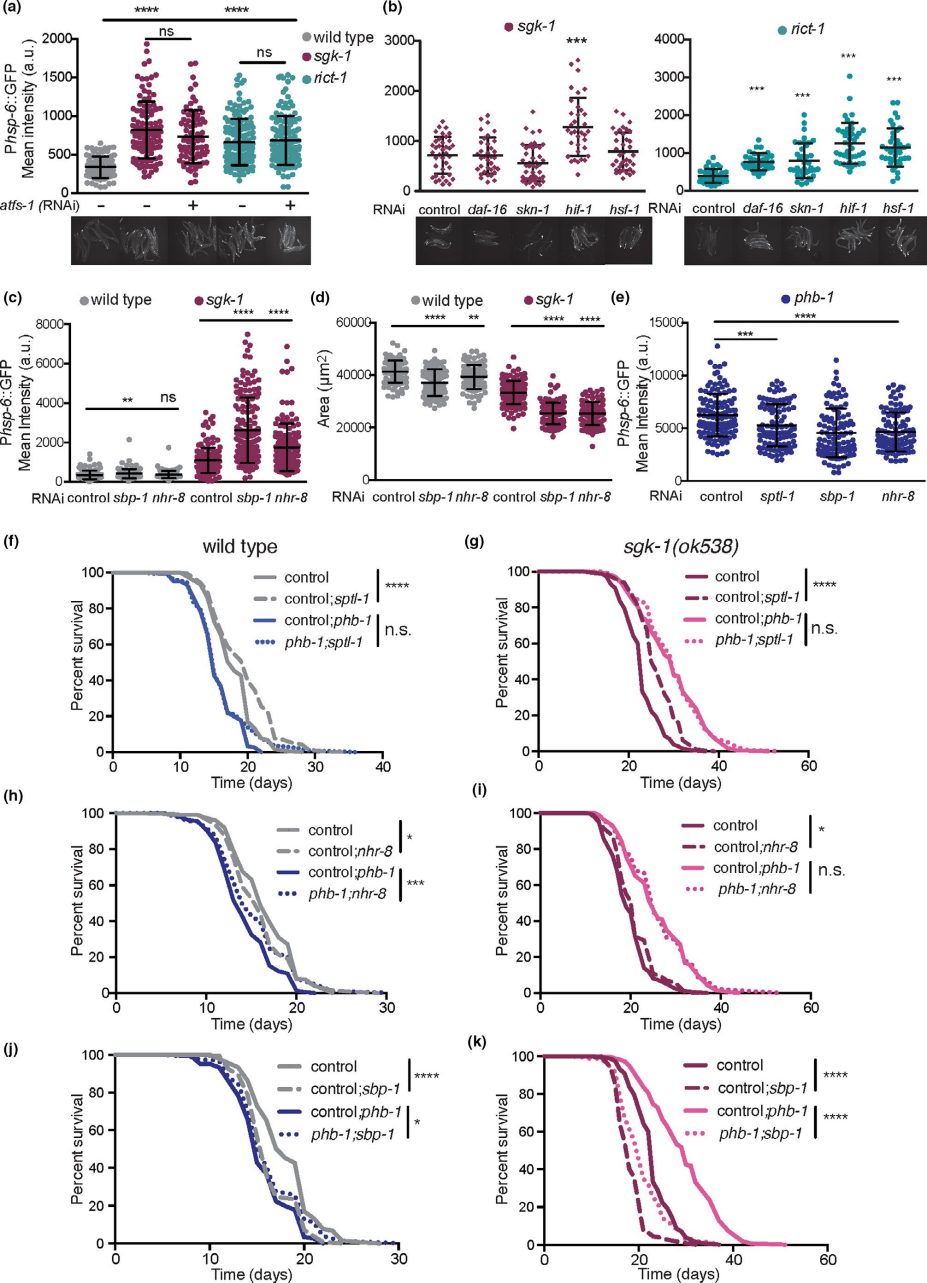
Altered sterol and lipid homeostasis mimic and exacerbate the UPR^mt^ induction of TORC2/SGK‐1 mutants. (a, b) Quantification of the UPR^mt^ reporter, P*hsp*‐*6*::GFP, in wild‐type animals, *sgk*‐*1* mutants and *rict*‐*1* mutants upon depletion of *atfs*‐*1* (a) and upon depletion of *daf*‐*16*, *skn*‐*1*, *hif*‐*1* and *hsf*‐*1* (b). Representative images are shown. (c) Quantification of the UPR^mt^ reporter in wild‐type animals and *sgk*‐*1* mutants upon depletion of *sbp*‐*1* and *nhr*‐*8*. (d) Quantification of worm area in wild‐type animals and *sgk*‐*1* mutants upon depletion of *sbp*‐*1* and *nhr*‐*8*. (e) Quantification of the UPR^mt^ reporter in *phb*‐*1(RNAi)* animals upon depletion of *sptl*‐*1*, *sbp*‐*1* and *nhr*‐*8*. Double RNAi was applied from hatching. Mean ± *SD* from three independent biological replicates; ****p < 0.0001, ***p < 0.001, **p < 0.05, *p < 0.1, ns not significant compared against control(RNAi); ANOVA test. (f–k) Lifespan analysis of wild‐type animals, *phb*‐*1(RNAi)* treated worms, *sgk*‐*1* mutants and *sgk*‐*1*;*phb*‐*1* depleted animals upon depletion of *sptl*‐*1* (f and g) *nhr*‐*8* (h and i) and *sbp*‐*1* (j and k). Double RNAi was applied from the L3 larval stage. Lifespan replicates and statistics are shown in Table S1

To identify additional pro‐survival pathways responsible for the long lifespan observed in PHB‐depleted TORC2/SGK‐1 mutants (Gatsi et al., 2014), we looked for transcription factors mediating the UPR^mt^ in the TORC2 mutants *rict*‐*1*(*ft17*) and *sgk*‐*1*(*ok538*). Surprisingly, ATFS‐1, a key transcription factor for UPR^mt^ induction (Nargund et al., 2012) was not required for P*hsp*‐*6*::GFP expression in the TORC2/SGK‐1 mutants (Figure 2a). In fact, while ATFS‐1 fully mediates the UPR^mt^ induction of PHB‐deficient animals (Hernando‐Rodriguez et al., 2018; Figure S2a), *rict*‐*1* and *sgk*‐*1* mutants depleted of PHB retained higher levels of *hsp*‐*6* induction upon *atfs*‐*1*(*RNAi*) than PHB‐depleted animals (Figure S2a). Transcription factors previously reported to be involved in maintenance of mitochondrial homeostasis and lifespan, DAF‐16, SKN‐1, HIF‐1 and HSF‐1 (Labbadia et al., 2017; Lee et al., 2010; Palikaras et al., 2015), were also analysed. Lack of HIF‐1 further induced the UPR^mt^ in *sgk*‐*1* mutants, while in *rict*‐*1* mutants depletion of all the transcription factors further enhanced the UPR^mt^ (Figure 2b), suggesting that TORC2 deficiency modulates the UPR^mt^ through other kinases in addition to SGK‐1. However, none of the transcription factors was required for the expression of *hsp*‐*6* in TORC2/SGK‐1 mutants.

In order to identify new transcription factors related to mitochondrial homeostasis in *sgk*‐*1* mutants, we knocked down by RNAi 836 transcription factors of the *C. elegans* genome and quantified the expression of the UPR^mt^ reporter. When grown in bacterial liquid cultures, we found 20 genes whose depletion reduced the UPR^mt^ in *sgk*‐*1* mutants (FC < 0.6; Figure S2b). Among those, we focused on genes that when depleted reduced worm size, as a proxy for genetic interaction (Figure S2c). We concentrated on NHR‐8 and SBP‐1, both involved in cholesterol and lipid homeostasis. NHR‐8 regulates cholesterol levels, fatty acid desaturation and apolipoprotein production (Magner et al., 2013). SBP‐1, the homologue of sterol regulatory element‐binding protein SREBP‐1, is required to produce cholesterol and to regulate the expression of lipogenic genes across phyla (Walker et al., 2011). Interestingly, when transcription factors were re‐tested on plate, the induction of the UPR^mt^ behaved in an opposing manner than in the RNAi screen (where worms were grown in liquid conditions) being the UPR^mt^ further increased in *sgk*‐*1* mutants upon *nhr*‐*8* or *sbp*‐*1* depletion, (Figure 2c). One possibility is that TORC2/SGK‐1‐mediated physiological responses are sensitive to growing conditions (Mizunuma et al., 2014). Since liquid growing conditions are energetically more demanding, the metabolism might be altered and the UPR^mt^ differentially regulated in *sgk*‐*1* mutants. Understanding the underlying biological mechanism behind this differential behaviour deserves further investigation. Nevertheless, identifying interactions between genes (by enhancing or suppressing a certain phenotype) shows that they functionally interact to regulate the same process. Loss of function of SGK‐1 reduces worm size, slows down development (Jones et al., 2009; Soukas et al., 2009) and induces the UPR^mt^ (Gatsi et al., 2014) and all phenotypes were exacerbated upon depletion of SBP‐1 and NHR‐8 (Figure 2c,d). In addition, depletion of NHR‐8 or SBP‐1 specifically slowed down the developmental rate of *sgk*‐*1* mutants but not of wild‐type animals (Figure S2d), indicating a synthetic interaction.

Apart from sterol (Roelants et al., 2018), ceramide and sphingolipids are key structural lipids of membranes and are regulated by Ypk1/SGK‐1 in yeast (Muir et al., 2014; Niles et al., 2014). We targeted by RNAi *sptl*‐*1*, a serine palmitoyl‐CoA acyltransferase responsible for the first committed step in de novo sphingolipid synthesis, and *cgt*‐*3*, a ceramide glucosyltransferase, previously shown to interact with SGK‐1 (Zhu et al., 2015). Depletion of SPTL‐1 and CGT‐3 induced the UPR^mt^ in otherwise wild‐type worms, similar to the *sgk*‐*1* mutant phenotype (Figure S2e). As reported, *cgt*‐*3* depletion slowed down development in *sgk*‐*1* mutants (Figure S2f; Zhu et al., 2015) and only 10%–20% of animals reached adulthood, while the rest had arrested development as L3 larvae. Depletion of *sptl*‐*1* slightly slowed down development and caused a partial (10 to 15%) larval arrest in otherwise wild‐type animals, while in *sgk*‐*1* 100% of the mutants were arrested at the L3 stage (Figure S2f). Thus, the process of ceramide and sphingolipids synthesis synthetically interacts with *sgk*‐*1* deletion. As the double mutants had arrested development, the *hsp*‐*6* expression levels could not be compared, however, depletion of *sptl*‐*1* and *cgt*‐*3* seemed to further induce the UPR^mt^ in *sgk*‐*1* mutants (Figure S2f). These results together suggest that defective lipogenesis and/or altered cholesterol and sphingolipid metabolism cause mitochondrial stress. Furthermore, the induced UPR^mt^ observed in *sgk*‐*1* mutants may result from altered lipid and sterol homeostasis.

### 2.3 Upon mitochondrial stress, lipid homeostasis interacts with TORC2/SGK‐1 to modulate the UPR^mt^ and to determine lifespan

To further understand the role of lipid metabolism in the PHB/SGK‐1 interaction, we tested the role of sphingolipids (*sptl*‐*1*) and sterol sensing transcription factors (*sbp*‐*1* and *nhr*‐*8*) in the UPR^mt^ and in lifespan. Depletion of all three lipid metabolism genes reduced the UPR^mt^ in PHB‐depleted animals (Figure 2e), mimicking the effect of *sgk*‐*1* deletion (Gatsi et al., 2014). While *sptl*‐*1* has already been shown to be required for the homeostatic response to mitochondrial defects caused by other paradigms (Liu et al., 2014), *sbp*‐*1* and *nhr*‐*8* have never been implicated in the UPR^mt^. In order to test the requirement of lipid metabolism genes for the UPR^mt^ in *sgk*‐*1*;*phb*‐*1(RNAi)* treated mutants, RNAi was applied from the late‐L3 larval stage given the interaction of lipid metabolism genes with *sgk*‐*1* during development. In this case, only *nhr*‐*8* was required for the UPR^mt^ response (Figure S2g). It remains, however, to be tested if a longer RNAi exposure could reveal a role for *sptl*‐*1* and *sbp*‐*1* in modulating the UPR^mt^ in *sgk*‐*1* mutants upon mitochondrial stress. Alternatively, *spb*‐*1* and *sptl*‐*1* might function during early development to trigger a proper UPR^mt^. In fact, in PHB‐depleted animals, late depletion of lipid metabolism genes revealed the involvement of *nhr*‐*8*, while early depletion (L1 larval stage) showed the requirement of all three genes, *nhr*‐*8*, *sbp*‐*1* and *sptl*‐*1*. (Figure S2g vs. Figure 2e). The reduced UPR^mt^ upon depletion of lipid genes under mitochondrial stess reminds the differential phenotype observed in *sgk*‐*1* mutants in liquid (RNAi screen) versus solid growing conditions. Thus, it is possible that growing on liquid possesses additional mitochondrial stress to *sgk*‐*1* mutants.

We performed lifespan analysis to determine the implication of sphingolipids and sterol sensing transcription factors in PHB‐mediated lifespan extension. Reduced sphingolipid synthesis in *sptl*‐*1*(*RNAi*) animals did not affect the lifespan of PHB‐depleted animals while it extended that of wild‐type animals as reported (Cutler et al., 2014; Figure 2f; Table S1). Surprisingly, despite their synthetic lethal interaction during development, the postdevelopmental depletion of *sptl*‐*1* extended the lifespan of *sgk*‐*1* mutants (Figure 2g; Table S1), while the lifespan extension conferred by PHB depletion was not affected (Figure 2g; Table S1). Thus, lifespan extension upon *sptl*‐*1* depletion requires a functional PHB complex. Regarding sterol sensing transcription factors, *nhr*‐*8* depletion shortened wild‐type lifespan as previously shown (Thondamal et al., 2014; Figure 2h) while it increased the lifespan of *phb*‐*1(RNAi)* animals and *sgk*‐*1* mutants (Figure 2h,i) without affecting the lifespan extension conferred by PHB depletion (Figure 2i). Depletion of *sbp*‐*1* shortened wild‐type lifespan as previously reported (Lee et al., 2015) and, similar to *nhr*‐*8* depletion, it also increased the lifespan of *phb*‐*1(RNAi)* treated animals (Figure 2j). Interestingly, *sbp*‐*1* depletion shortened the lifespan of *sgk*‐*1* mutants more than that of wild‐type animals (Figure 2j,k; 21.7% vs. 11.1%) and even more that of PHB‐depleted *sgk*‐*1* mutants (30%; Figure 2k). Thus, regulation of lipid metabolism by SBP‐1 is required for the long lifespan of *sgk*‐*1* mutants as well as for the further lifespan extension conferred by PHB depletion. The lack of correlation between UPR^mt^ and lifespan effects supports the conclusion that the different lipid metabolism genes modulate the UPR^mt^ and longevity in an independent manner. Interestingly, *nhr*‐*8* and *sbp*‐*1* are required for PHB depletion phenotypes, UPR^mt^ induction and lifespan shortening. However, only *sbp*‐*1* was partially required for the increased lifespan conferred by PHB depletion to *sgk*‐*1* mutants. Supporting the role of sterols in the UPR^mt^, cholesterol supplementation reduced the UPR^mt^ in PHB‐depleted conditions (Figure S2h). The effect of cholesterol treatment in the expression of sterol sensing transcription factors, as well as in mitochondrial stress resistance and longevity remains to be determined.

### 2.4 Prohibitin depletion suppresses lipogenesis and lipoprotein/yolk formation defects of *sgk*‐*1* mutants

SBP‐1 undergoes an export from the ER to Golgi, in response to altered membrane lipid ratios (Smulan et al., 2016). In mammals, mTORC2 and SGK1 have been shown to regulate mitochondrial function by maintaining mitochondria‐associated ER membrane (MAM) integrity (Betz et al., 2013), required for synthesis and maturation of key membrane lipids including cholesterol, phospholipids and sphingolipids (Vance, 2014).

**FIGURE 3:**
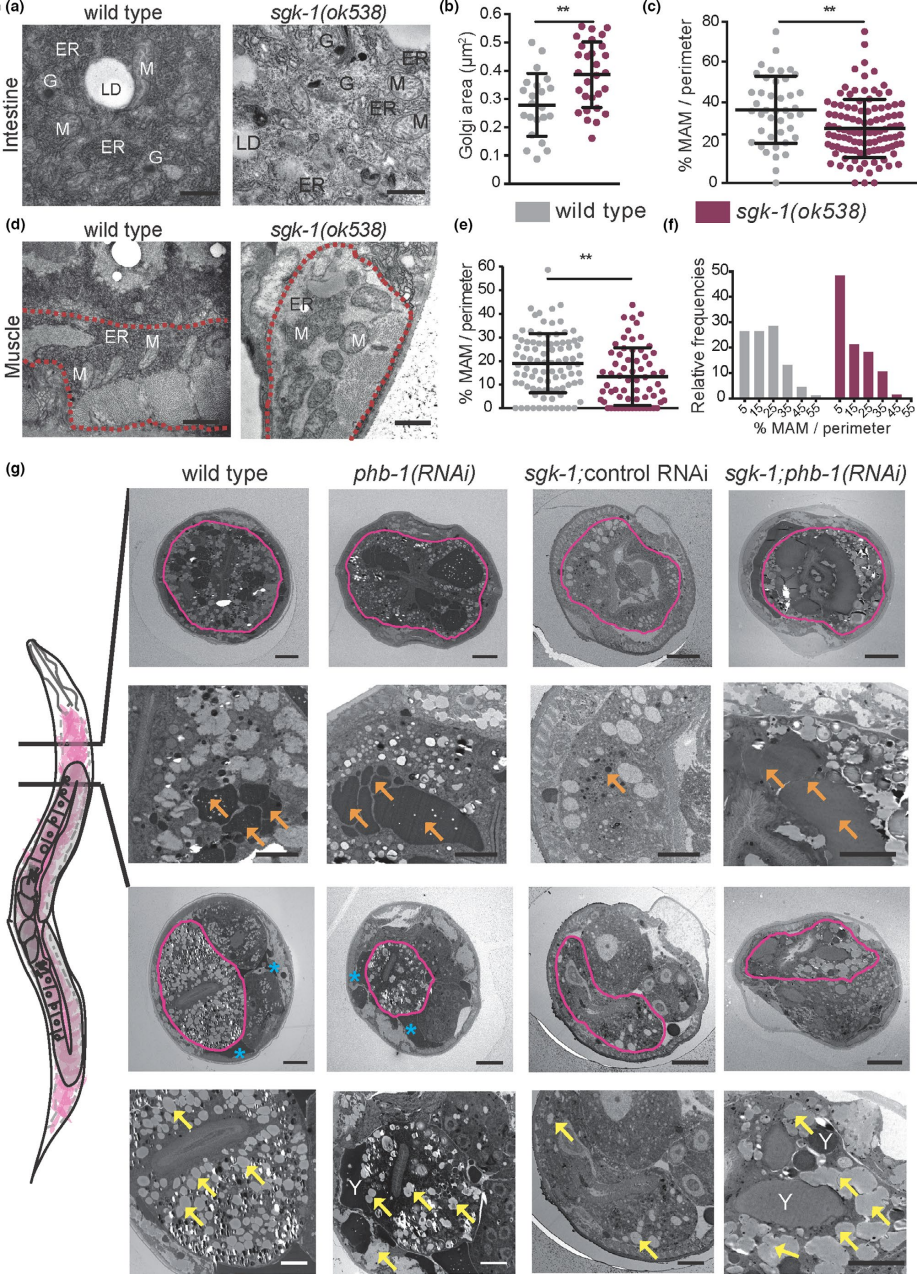
Prohibitin depletion suppresses lipogenesis and lipoprotein/yolk formation defects of *sgk*‐*1* mutants. (a) Transmission electron microscopy (TEM) images of the first intestinal cells of wild‐type and *sgk*‐*1* mutants at day one of adulthood. Bar size: 500 nm. ER: Endoplasmic Reticulum, LD: Lipid Droplet, Y: Yolk, G: Golgi. (b) Quantification of Golgi areas in intestine of wild‐type and *sgk*‐*1* mutants at day one of adulthood. (c) Quantification of the ER‐mitochondria contact site length in intestine, normalized to mitochondrial perimeter, of wild‐type and *sgk*‐*1* mutants, at day one of adulthood. Mean ± *SD*; **p < 0.05; *t* *test*, *n* > 40 contact sites. (d) TEM images of muscle, delineated with a red dashed line, of wild‐type and *sgk*‐*1* mutants at day one of adulthood. Bar size: 1 μm. M: Mitochondria. (e) Quantification of the ER‐mitochondria contact site length in muscle, normalized to mitochondrial perimeter, of wild type and *sgk*‐*1* mutants, at day one of adulthood. Mean ± *SD*; **p < 0.05; *t*‐*test*, *n* > 60 contact sites. (f) Frequency distribution histogram, as per cent, from data set shown in panel e, bin width 10. (g) TEM images of wild type, *sgk*‐*1* mutants, *phb*‐*1* depleted animals and *sgk*‐*1*;*phb*‐*1(RNAi)* treated mutants at day five of adulthood. Two sections, before and after the gonad turn are shown. Top images show a general view where the intestine is delineated with a pink line. Bottom images show a magnification of the intestinal area. Bar sizes: 10 μm (top panels) and 5 μm (bottom panels) for each of the cuts. Orange arrows mark yolk, blue asterisks mark pseudocoelomic lipoproteins, yellow arrows label LD, Y: yolk

We used TEM to explore the Golgi apparatus and the ER in *sgk*‐*1* mutants. At day 1 of adulthood, in intestinal cells, we observed an increased size of the Golgi system in *sgk*‐*1* mutants, where more cisternae and vesicles were visible (Figure 3a,b, highlighted in Figure S3a). This suggests that Golgi functionality, and probably Golgi‐ER communication, is altered in *sgk*‐*1* mutants. We next analysed the role of SGK‐1 in maintaining regions of contact between the ER and mitochondria by measuring MAM. In the intestine, MAMs were reduced in *sgk*‐*1* mutants as compared to wild type (Figure 3c and visible in Figure 3a; Figure S3b). Similarly, in muscle cells, *sgk*‐*1* mutants showed reduced MAM, with some areas completely devoid of ER (Figure 3d,e, also visible in Figure S1a). Compared to wild type, *sgk*‐*1* mutants showed a higher proportion of mitochondria with smallest percentages in contact with ER (Figure 3f). The altered Golgi structure and ER‐mitochondrial interaction suggest that organellar contact sites might be generally affected in *sgk*‐*1* mutants. Interaction of lipid droplets with vacuoles also seemed to be affected in *sgk*‐*1* mutants (Figure S3b). At day five of adulthood, large lipid droplets accumulate in the intestine of wild‐type animals, while in *sgk*‐*1* mutants only small lipid droplets could be observed (Figure S3c). Defects in MAM, and other ER membrane contacts, could account for the impaired lipid droplet and yolk/lipoprotein accumulation previously reported in *sgk*‐*1* mutants (Dowen et al., 2016; Wang et al., 2016; Yen et al., 2010) and also observed here (see below, Figure 3g). Importantly, *sgk*‐*1* mutants showed the above‐described phenotypes; increased mitochondrial size and reduced lipid and yolk content, also when fed on OP50, a different bacterial food source (Figure S3d).

We analysed TEM sections in the anterior of the intestine, before and after the gonad turn, in five‐days old animals, where significant amounts of yolk (orange arrows) and lipid droplets (yellow arrows) accumulate in wild‐type animals (Figure 3g). Defects in vitellogenesis/yolk accumulation and lipid droplet formation were obvious in *sgk*‐*1* mutants (Figure 3g). In addition, little accumulation of lipoprotein pools at the pseudocoelom (blue asterisks) was observed compared to wild‐type and PHB‐depleted animals (Figure 3g). Strikingly, depletion of the mitochondrial PHB complex suppressed both, lipid droplet accumulation and yolk production defects of *sgk*‐*1* mutants (Figure 3g).

### 2.5 Prohibitin depletion and *sgk*‐*1* deletion differentially induce autophagy and lysosomal function

**FIGURE 4:**
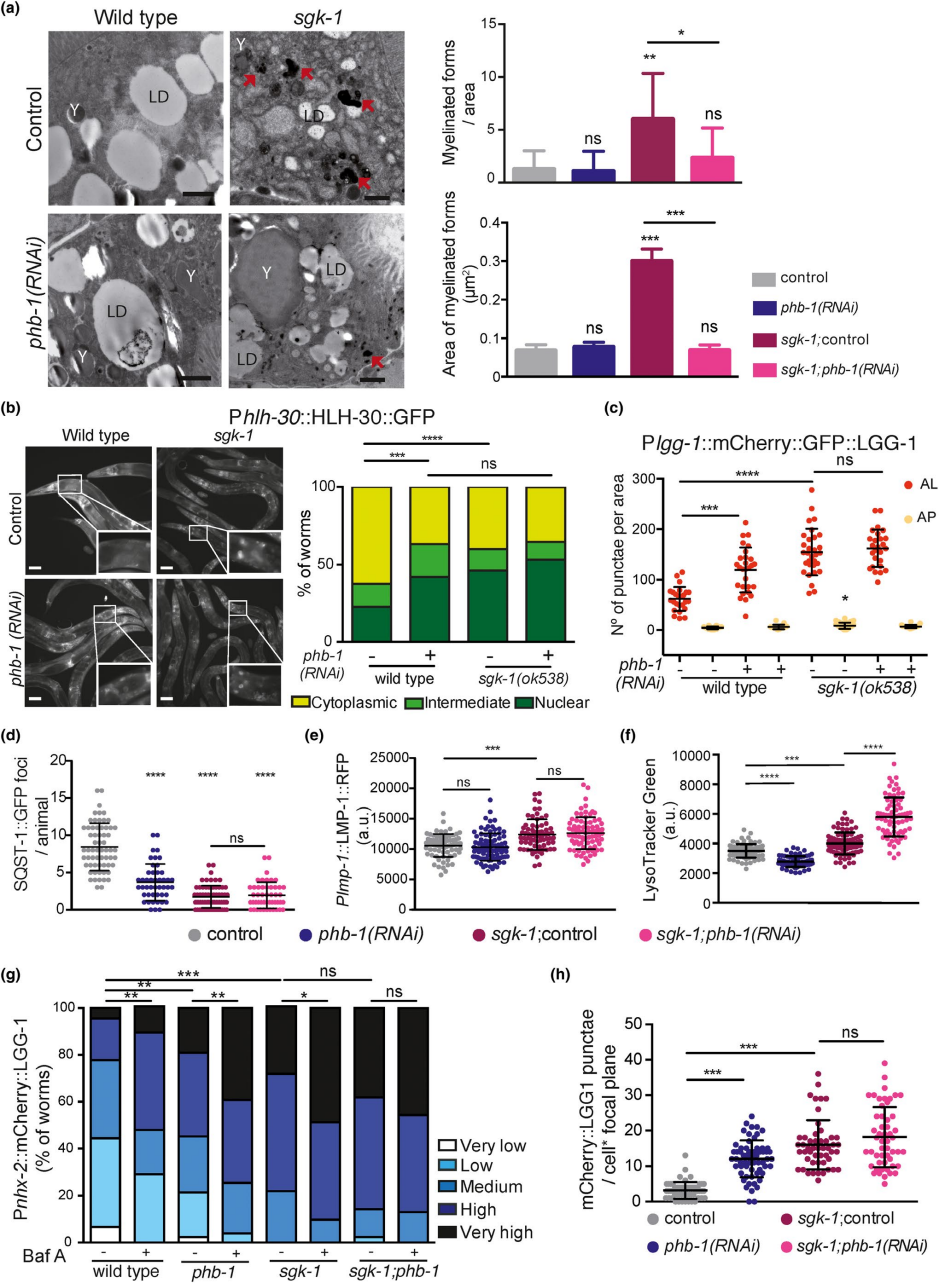
Prohibitin depletion and *sgk‐1* deletion differentially induce autophagy and lysosomal content. All analyses were performed in wild‐type animals, *phb*‐*1(RNAi)* worms, *sgk*‐*1* mutants and *sgk*‐*1*;*phb*‐*1(RNAi)* treated mutants, (a) Electron microscopy images at day five of adulthood. Red arrows show myelinated forms quantified in the right panel, both number and size. LD: lipid droplet, Y: yolk particles. Bar size: 1 μm. Mean SD; ***p < 0.001, **p < 0.05, *p < 0.5, ns not significant; *t*‐test. Combination of three independent replicates. (b) Images (left) and quantifications (right) of P*hlh*‐*30*::HLH‐30::GFP at day one of adulthood. Bar = 10 μm. White squares show zoomed in images of the first intestinal cells. Mean ± *SD* from at least four independent biological replicates; ****p < 0.0001, ***p = 0.0001, ns not significant; Mann–Whitney test. (c) Quantification of autophagosomes (AP) and autolysosomes (AL) in the first intestinal cells at day one of adulthood using the P*lgg*‐*1*::mCherry::GFP::LGG‐1 reporter. Representative images are shown in Figure S4b. Mean ± *SD* of three independent replicates; ****p < 0.0001, ***p = 0.0001, **p < 0.05, *p < 0.5; ns not significant; ANOVA test. (d) Quantification of the autophagy substrate SQST‐1 in the head of day one adults. Representative images are shown in Figure S4e. Mean ± *SD* of three independent replicates; ****p < 0.0001, ns not significant; ANOVA test. (e) Quantification of the lysosomal maker P*lmp*‐*1*::LMP‐1::RFP at day one of adulthood. Mean ± *SD* of at least four independent replicates; ***p = 0.0001, ns not significant; ANOVA test. Representative images are shown in Figure S4f. (f) Quantification of the lysosomal specific dye Lysotracker Green at day one of adulthood. Mean ± *SD* of three independent replicates; ****p < 0.0001, ***p = 0.0001; ANOVA test. (g) Quantification of the intestinal autophagy reporter Ex[P*nxh*‐*2*::mCherry::LGG‐1] in animals treated or not with bafilomycin A1 (Baf A), at day five of adulthood. ***p < 0.0001, **p < 0.01, *p < 0.05, ns not significant; Mann–Whitney test. Data from two biological replicas using between 16 and 29 worms per condition in each replica. Representative images are shown in Figure S4i. (h) Quantification of the intestinal integrated autophagy reporter *byIs205* [P*nxh*‐*2*::mCherry::LGG‐1]. Mean ± *SD* of three independent replicates; ***p < 0.001, ns not significant; ANOVA test. Representative images are shown in Figure S4k

MAMs participate in lipid synthesis (Vance, 2014) and provide membranes for autophagosome formation (Hamasaki et al., 2013). Moreover, autophagy is required for normal lipid levels (Lapierre, Silvestrini, et al., 2013) and for the conversion of intestinal lipids into yolk (Ezcurra et al., 2018). In metazoans, SGK‐1 inhibits autophagy (Aspernig et al., 2019; Zhou et al., 2019). In our TEM analysis, we observed complex structures containing myelinated membranes in *sgk*‐*1* mutants at day one (Figure S3b–d) and at day five of adulthood (Figure 4a, red arrows and Figure S4a). These lysosome‐related structures could originate from the endocytic pathway or from autolysosomes. Quantification of such structures showed that PHB depletion suppressed the accumulation of myelinated forms in *sgk*‐*1* mutants, as well as their size (Figure 4a). The accumulation of dark autolysosomal compartments can be the consequence of impaired lysosomal digestion (Zhang et al., 2015), raising the possibility that cargo degradation is hindered in *sgk*‐*1* mutants and suppressed by PHB depletion.

To better understand the interaction of PHB and SGK‐1 in autophagy in *C. elegans*, we looked at the subcellular localization of the transcription factor HLH‐30/TFEB (Lapierre, De Magalhaes Filho, et al., 2013), a positive regulator of autophagy and lysosome biogenesis (Settembre et al., 2011), in the first intestinal cells. Compared to wild type, *sgk*‐*1* deletion stimulated HLH‐30 nuclear localization (Figure 4b). In a different allele, *sgk*‐*1(mg455)*, HLH‐30 protein levels were shown to increase (Zhou et al., 2019). Similarly, PHB depletion induced the nuclear localization of HLH‐30, while no additive effect was observed in *sgk*‐*1*; *phb*‐*1* depleted worms (Figure 4b). We monitored autophagosomes (APs) and autolysosomes (ALs) using a tandem‐tagged mCherry::GFP::LGG‐1 reporter (Chang et al., 2017). Under the acidic environment of ALs, GFP is quenched, therefore, ALs appear as red punctae, while APs appear as yellow [green and red] punctae. The number of intestinal APs slightly increased only in *sgk*‐*1* mutants compared with wild type (Figure 4c). However, the number of ALs increased considerably in PHB‐depleted animals and in *sgk*‐*1* mutants, while no additive effect was observed upon PHB depletion in *sgk*‐*1* mutants (Figure 4c; Figure S4b). The number of ALs was also increased in the intestine and hypodermis of *sgk*‐*1* mutants when grown in OP50 bacteria (Figure S4c,d). In accordance, clearance of the autophagy target SQST‐1/p62 (Tian et al., 2010) was increased in PHB‐depleted animals and in *sgk*‐*1* mutants (Figure 4d; Figure S4e), as previously reported (Aspernig et al., 2019), Reduction of SQST‐1::GFP punctae was not enhanced by PHB depletion in *sgk*‐*1* mutants (Figure 4d; Figure S4e). Those phenotypes suggested increased autophagy upon PHB depletion and in *sgk*‐*1* mutants, with no further alterations in *sgk*‐*1*; *phb*‐*1(RNAi)*‐depleted animals. However, surprisingly, only *sgk*‐*1* mutants showed increased levels of the RFP‐tagged lysosomal protein LMP‐1 (Campbell & Fares, 2010) in intestinal cells, which were not further affected by PHB depletion (Figure 4e; Figure S4f). This indicates that ALs observed in *phb*‐*1(RNAi)* animals are LMP‐1 negative or LMP‐1 might be labelling intestinal endolysosomes rather than autolysosomes. However, when animals were stained using a lysosome‐specific fluorescent dye, PHB depletion reduced lysosomal content in wild‐type animals while it further increased the staining of *sgk*‐*1* mutants (Figure 4f; Figure S4g). This increased staining was not associated with autofluorescent granules (Figure S4h). Further studies are required to determine the nature of LysoTracker Green‐labelled structures using other intestinal granule markers.

To assess autophagic flux, we used Bafilomycin A1 (BafA), an inhibitor of lysosomal acidification that prevents autophagic turnover. We looked at day 5 of adulthood animals using the extrachromosomal intestinal autophagy marker mCherry::LGG‐1 which results in a punctuated expression pattern upon LGG‐1 recruitment to autophagosomes (Gosai et al., 2010). We classified the level of autophagy based on 5 different categories (see Materials and Methods and Figure S4i). At day 5 of adulthood, *sgk*‐*1* mutants showed an enhanced autophagy signal compared with wild‐type animals, which was reduced upon *unc*‐*51* depletion (Figure S4j), an essential gene for autophagosome formation. Upon BafA treatment, wild‐type worms showed increased LGG‐1 punctae (Figure 4g). BafA also increased LGG‐1 punctae in PHB‐depleted animals and in *sgk*‐*1* mutants (Figure 4g). However, the increase in *sgk*‐*1* mutants was lower than the one observed in wild‐type worms (11% vs 34%). Surprisingly, *sgk*‐*1* mutants depleted of PHB did not show an increase in LGG‐1 punctae upon BafA treatment (Figure 4g). Although these data suggest a blockage of the autophagic flux, the results are not conclusive, as BafA diffusion could differ between genetic backgrounds due to differences in membrane lipid composition. To ensure that the extrachromosomal marker correctly reflects LGG‐1 punctae, we used an integrated version of the array (Aspernig et al., 2019) and similar results were observed; increased LGG‐1 punctate pattern in PHB‐depleted worms and in *sgk*‐*1* mutants (as previously reported, Aspernig et al., 2019), with no further increase in *sgk*‐*1*;*phb*‐*1(RNAi)*‐depleted animals (Figure 4h and Figure S4k). In sum, our data suggest that autophagy is triggered through the same molecular pathway in response to SGK‐1 and PHB deficiency, since no additive effect is observed in the double mutant. However, lysosomal function seems to be differentially regulated and additional studies are required.

### 2.6 Prohibitin depletion increases longevity of *sgk*‐*1* mutants through autophagy and the UPR^mt^, but not mitophagy

**FIGURE 5:**
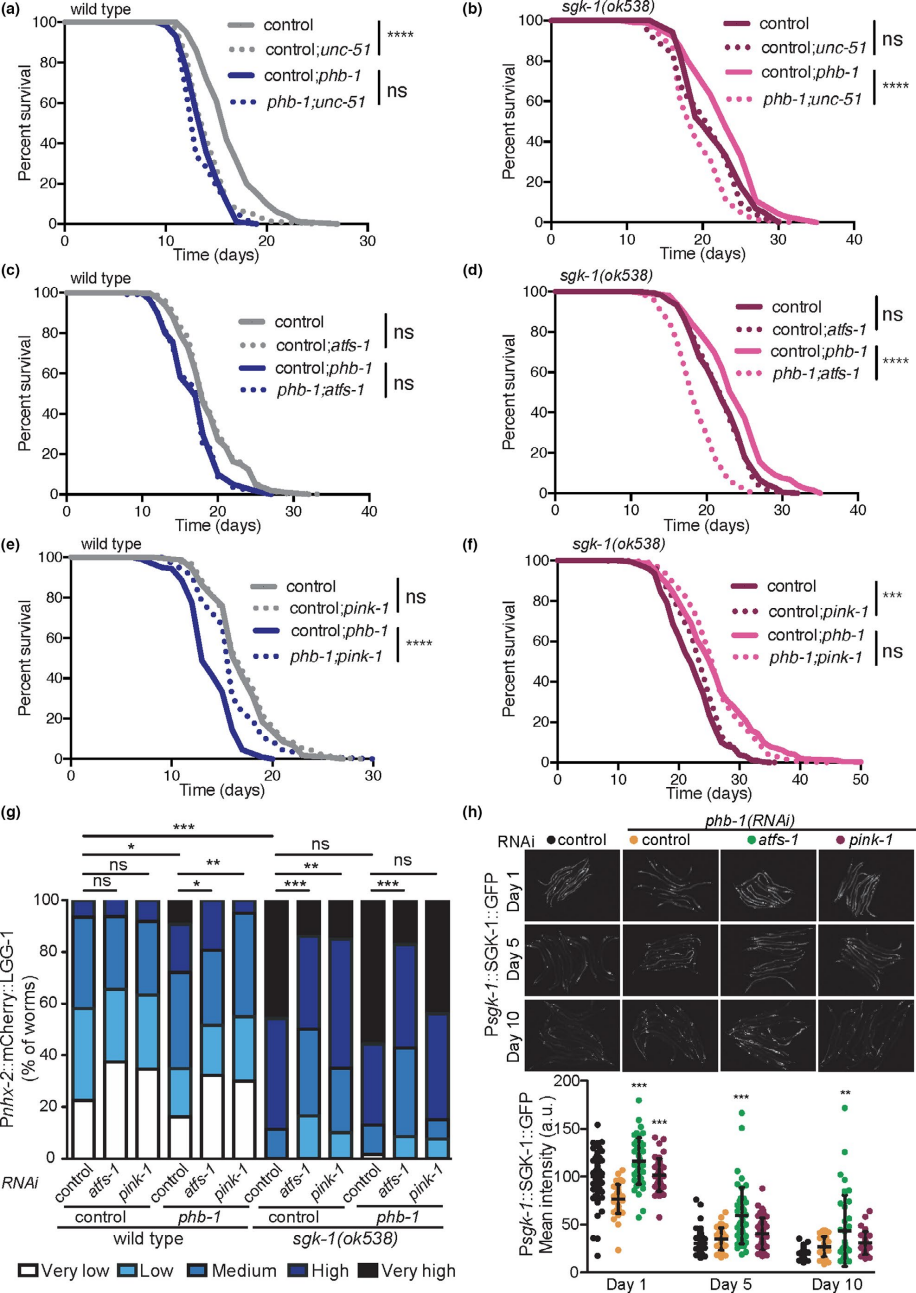
Autophagy and the UPR^mt^, but not mitophagy, are required for the enhanced longevity of *sgk*‐*1* mutants upon PHB depletion. (a–f) Lifespan of wild‐type worms (left) and *sgk*‐*1* mutants (right) with or without the PHB complex (*phb*‐*1(RNAi*)) upon inhibition of autophagy (a and b), inhibition of the UPR^mt^ (c and d) and inhibition of mitophagy (e and f). Replicates and statistics are shown in Table S1. (g) Quantification of the autophagy reporter Ex[P*nxh*‐*2*::mCherry::LGG‐1] in wild‐type animals, *phb*‐*1(RNAi)* treated worms, *sgk*‐*1(ok538)* mutants and *sgk*‐*1*;*phb*‐*1* depleted mutants at day 5 of adulthood upon inhibition of the UPR^mt^ (*atfs*‐*1(RNAi)*) and mitophagy (*pink*‐*1(RNAi)*). ***p < 0.001, **p < 0.01, *p < 0.1, ns not significant; Mann–Whitney test. Combination of at least three independent replicates. (h) SGK‐1 protein levels during ageing, at day 1, 5 and 10 of adulthood, upon PHB depletion and inhibition of either the UPR^mt^ (*atfs*‐*1(RNAi)*) or mitophagy (*pink*‐*1(RNAi)*). Mean ± *SD*; ***p < 0.001, **p < 0.01, *p < 0.1, ns not significant; ANOVA test. Combination of at least three independent replicates is shown

Long‐lived *sgk*‐*1(ok538)* mutants and PHB‐depleted animals have induced mitochondrial quality control mechanisms such as mitophagy (Figure S5a; Aspernig et al., 2019), the UPR^mt^ (Gatsi et al., 2014) and autophagy (Figure 4; Aspernig et al., 2019). Therefore, we investigated the requirement of autophagy genes as well as both mitochondrial quality control mechanisms, UPR^mt^ and mitophagy, for the lifespan extension conferred by PHB depletion to *sgk*‐*1* mutants. Inhibiting autophagy by *unc*‐*51*(*RNAi*) treatment did not affect the lifespan of *phb*‐*1* depleted worms nor of *sgk*‐*1* mutants while it shortened that of *sgk*‐*1*;*phb*‐*1(RNAi)* treated animals and wild‐type worms (Figure 5a,b; Table S1). Likewise, inhibition of autophagy initiation (*unc*‐*51*(*RNAi*)), inhibition of nucleation (*bec*‐*1*(*RNAi*)) and elongation (*atg*‐*16*(*RNAi*)) shortened the lifespan of the *sgk*‐*1*;*phb*‐*1*(*RNAi*) treated mutants (Figure S5b). Depletion of ATFS‐1, the key transcription factor in UPR^mt^ activation, did not affect the lifespan of wild‐type animals, *sgk*‐*1* mutants or *phb*‐*1* depleted animals (Figure 5c,d; Table S1). However, preventing the UPR^mt^ suppressed the longevity of *sgk*‐*1*;*phb*‐*1(RNAi)* treated mutants down to wild‐type levels (Figure 5d; Table S1). We conclude that both mechanisms, autophagy and UPR^mt^, are required for the enhanced longevity of *sgk*‐*1* mutants upon PHB depletion.

We then assessed the requirement of mitophagy for lifespan in the different genetic backgrounds. Depletion of the mitochondrial phosphatase and tensin (PTEN)‐induced kinase 1 (PINK‐1) did not affect the lifespan of wild‐type worms (Figure 5e; Table S1), as previously described (Palikaras et al., 2015). Interestingly, preventing mitophagy increased the lifespan of PHB‐depleted worms and *sgk*‐*1* mutants (Figure 5e,f; Table S1) as reported for a different *sgk*‐*1* allele (Zhou et al., 2019). However, in *sgk*‐*1*;*phb*‐*1*(*RNAi*) treated worms inhibition of mitophagy did not affect lifespan (Figure 5f; Table S1). Furthermore, depletion of DCT‐1, a mitophagy receptor acting downstream of PINK‐1 (Palikaras et al., 2015), also increased the lifespan of PHB‐depleted worms and *sgk*‐*1* mutants (Figure S5c,d; Table S1) without affecting wild‐type worms (Figure S5c; Table S1), recapitulating *pink*‐*1(RNAi)* phenotypes. In the case of the *sgk*‐*1*;*phb*‐*1(RNAi)* treated mutants, *dct*‐*1* depletion mildly shortened lifespan (Figure S5d; Table S1). These results show that mitophagy can be detrimental in certain pre‐conditioned mutants such as *sgk*‐*1* and PHB‐depleted worms. To better understand the differential requirement of both mitochondrial quality control mechanisms, UPR^mt^ and mitophagy, for the lifespan extension of *sgk*‐*1* mutants upon PHB depletion, we examined their contribution to the pool of autophagosomes. Inhibiting the UPR^mt^ reduced the autophagic signal under all tested conditions (Figure 5g), as expected since ATFS‐1 directly regulates LGG‐1 expression (Nargund et al., 2015). Treatment with *pink*‐*1(RNAi)* reduced the accumulation of autophagosomes in *phb*‐*1(RNAi)* and in *sgk*‐*1* mutants but not in *sgk*‐*1*;*phb*‐*1(RNAi)* treated animals (Figure 5g).

We then tested the requirement of SGK‐1 in situations of mitochondrial stress and upon inhibition of the UPR^mt^ or mitophagy. In PHB‐depleted animals, inhibition of the UPR^mt^ resulted in a remarkable increase of intestinal SGK‐1 protein levels during ageing, while mitophagy inhibition did not (Figure 5h). Similar results were obtained when we pharmacologically induced mitochondrial stress with paraquat (PQ; Figure S5e). Thus, in the absence of ATFS‐1, SGK‐1 becomes more important to maintain mitochondrial/cellular homeostasis. Therefore, it is tempting to speculate that the mechanism by which PHB depletion extends *sgk*‐*1* mutant lifespan is by inducing the UPR^mt^, which in turn induces general autophagy and modulates mitochondrial metabolism (Nargund et al., 2015). In addition, ATFS‐1 could contribute to lifespan extension through the modulation of lipid metabolism, as it induces enzymes of the mevalonate/isoprenoid/cholesterol synthesis pathway upon mitochondrial stress (Oks et al., 2018; Rauthan et al., 2013).

## 3 DISCUSSION

Depletion of PHB, a multimeric ring‐like complex sitting in the inner mitochondrial membrane shortens lifespan in wild‐type worms. In contrast, in a *sgk*‐*1*‐mutant background, PHB depletion increases lifespan (Gatsi et al., 2014). By analysing the interaction between SGK‐1 and the PHB complex, we found that *sgk*‐*1* mutants have mitochondrial and lipogenic defects which are suppressed by PHB depletion. We have shown that while the lipogenic transcription factor SBP‐1 is partially required for the lifespan extension conferred by PHB depletion to *sgk*‐*1* mutants, it shortens the lifespan of PHB‐depleted wild‐type animals. We further demonstrated that autophagy and the UPR^mt^, but not mitophagy, are required for lifespan extension of *sgk*‐*1(ok538)*;*phb*‐*1(RNAi)* animals, but do not affect the lifespan of *sgk*‐*1(ok538)* or *phb1(RNAi)* animals. Our data suggest that PHB depletion induces the UPR^mt^, which promotes autophagy and probably balances membrane lipid defects of *sgk*‐*1* mutants, being beneficial for lifespan.

In *C. elegans*, we showed an unprecedented role for TORC2/SGK‐1 in the regulation of mitochondrial homeostasis. TORC2/SGK‐1 deficiency induced the UPR^mt^ and increased mitochondrial mass in the muscle and the intestine (Gatsi et al., 2014). More recently, in *C. elegans*, SGK‐1 has been shown to phosphorylate the voltage‐dependent anion channel VDAC1 (Zhou et al., 2019) and to maintain low mitochondrial‐derived ROS levels (Aspernig et al., 2019). The data we present here demonstrates a key role for SGK‐1 in endomembrane biology, which extends to mitochondrial integrity. By electron microscopy, we observed bigger mitochondria in *sgk*‐*1* mutants as well as reduced mitochondria‐associated ER membranes (MAM). Mitochondria in *sgk*‐*1* mutants appeared larger and hyperfused, resembling the phenotype observed in sphingolipid deficient (*sptl*‐*1*(*RNAi*)) worms (Liu et al., 2014) and supporting a role for SGK‐1 in membrane lipid homeostasis. We did not observe any obvious mitochondrial fragmentation by TEM as previously described (Zhou et al., 2019). Similarly, and contrary to what has been reported (Zhou et al., 2019), we observed an increase in oxygen consumption in *sgk*‐*1* mutants. Our data, however, agree with the increased mitochondrial respiration rate observed in mTORC2/rictor knockdown in mammalian cells (Schieke et al., 2006).

The possible reasons for this discrepancy are discussed below. In this study, we used the deletion allele *ok538* (Hertweck et al., 2004), while Zhou et al. used the *mg455* allele, (Soukas et al., 2009; Zhou et al., 2019). *sgk*‐*1(ok538*) harbours a 852 bp deletion that removes most of the SGK‐1 kinase domain critical for SGK‐1 activity. Moreover, inactivation of *sgk*‐*1* by RNAi results in very similar phenotypes (Hertweck et al., 2004; Jones et al., 2009). In *sgk*‐*1*(*mg455*), a nonsense mutation results in an early stop that removes 178 residues including critical components of the kinase domain and the hydrophobic motif phosphorylation site (Soukas et al., 2009). Both are assumed to be strong loss‐of‐function/null alleles. At 20°C in HT115 bacteria, *mg455* is short lived (Zhou et al., 2019) while *ok538* is long lived (Gatsi et al., 2014). The difference between the experiments performed by Zhou et al. and ours is that we do not use FUdR, an inhibitor of DNA synthesis used to prevent progeny production. We have shown that FUdR suppresses the extended lifespan of *sgk*‐*1(ok538)* mutants when grown at 20°C (Gatsi et al., 2014). To our knowledge, *mg455* has never been tested in the absence of FUdR at 20°C, which will discern if the differences observed in terms of lifespan and oxygen consumption are due to the allele or due to the growing conditions. Since both alleles share defects in brood size, growth rate and body size (Soukas et al., 2009), the differences might solely depend on the growing conditions. Moreover, transgenic expression of wild‐type SGK‐1 (Aspernig et al., 2019) rescued the lifespan extension of *ok538* mutants (Figure S5f; Table S2) eliminating the possible effect of any background mutations.

TORC2/SGK‐1 mutants are sensitive to differential environmental inputs such as growth temperature and nutrients, affecting lifespan positively or negatively. Many longevity experiments have been performed using different *rict*‐*1* and *sgk*‐*1* alleles and RNAi under different conditions. What seems consistent, so far, is that in the presence of FUdR, deficient TORC2/SGK‐1 signalling shortens lifespan regardless of the food source when grown at 20°C, while at 25°C it shortens lifespan in OP50 but it extends lifespan in K12‐type bacterial strains (HT115 and BW25113; Alam et al., 2010; Chen et al., 2013; Mizunuma et al., 2014; Shin et al., 2020; Soukas et al., 2009; Xiao et al., 2013; Zhou et al., 2019). However, in the absence of FUdR, TORC2/SGK‐1 deficiency consistently extends lifespan regardless of food source and temperature (Evans et al., 2008; Gatsi et al., 2014; Hertweck et al., 2004; Mizunuma et al., 2014; Rahman et al., 2010; Shin et al., 2020; Soukas et al., 2009). FUdR inhibits mitochondrial DNA replication (Rooney et al., 2014). Thus, it is possible that additional mitochondrial stress could revert the ageing phenotype and other physiological responses of TORC2/SGK‐1 animals, which already have compromised mitochondrial function (Aspernig et al., 2019; Gatsi et al., 2014; Zhou et al., 2019). In agreement, FUdR partially suppresses the extended lifespan of mitochondrial mutants (Shin et al., 2020). Thus, it is possible that mild mitochondrial dysfunction contributes to the extended lifespan of *sgk*‐*1* mutants. However, this lifespan extension is *atfs*‐*1* independent, as *atfs*‐*1* induction is not sufficient to extend lifespan (Rauthan et al., 2013). Whether different levels of mitochondrial dysfunction can affect respiration in an opposite manner in TORC2/SGK‐1 mutants deserves further investigation.

Consistent with increased respiration, *sgk*‐*1* mutants have higher ROS levels, both cytosolic (this work) and mitochondrial (Aspernig et al., 2019). Previous work suggests that mitochondrial ROS in TORC2/SGK‐1 deficient animals play a mitohormetic role, differentially regulating mitochondrial function and signalling depending on the level of stress (Aspernig et al., 2019). Our data support that view, since increasing mitochondrial ROS in *sgk*‐*1* mutants in the presence or the absence of mitochondrial PHB have an opposing effect on lifespan. Interestingly, a link between ROS levels and sphingolipids homeostasis has been proposed for yeast TORC2/Ypk1, although the molecular bases are unknown (Niles et al., 2014). TORC2/Ypk1 activates sphingolipid and ceramide biosynthesis (Muir et al., 2014) and, in *C. elegans*, SGK‐1 has been proposed to regulate membrane trafficking through sphingolipids (Zhu et al., 2015). We show here that depletion of key genes in the synthesis of ceramides and sphingolipids induce the UPR^mt^ as *sgk*‐*1* deletion does (Gatsi et al., 2014) and synthetically interact with *sgk*‐*1*. Interestingly, in worms, inhibition of sphingolipids biosynthesis (*sptl*‐*1*(*RNAi*)) reduces induction of the UPR^mt^ upon antimycin treatment (Liu et al., 2014) and PHB depletion (Figure 2e) similar to the effect of *sgk*‐*1* deletion in PHB depleted animals (Gatsi et al., 2014). As low *sgk*‐*1* signalling does, *sptl*‐*1* depletion increases lifespan (Cutler et al., 2014) of wild‐type animals. Moreover, *sptl*‐*1* depletion in *sgk*‐*1* mutants, from the L3 stage, further extends lifespan, suggesting that *sgk*‐*1* modulates lifespan through additional mechanisms. Interestingly, PHB is required for the lifespan extension conferred by reduced sphingolipids. It is also possible that SGK‐1 signalling might differentially affect sphingolipid synthesis under mitochondrial stress versus non‐stress conditions.

Furthermore, our transcription factor RNAi screen uncovered an interaction of membrane sterol and lipid homeostasis with SGK‐1. Depletion of NHR‐8 or SBP‐1, further enhanced induction of the UPR^mt^ in *sgk*‐*1* mutants (Figure 2c). However, under mitochondrial stress conditions, the UPR^mt^ phenotype is reverted and depletion of lipogenic/sterol sensing transcription factors (TFs) suppress induction of the UPR^mt^ (Figure 2e). Again, suggesting a differential regulation depending on mitochondrial stress levels. We find no association between the UPR^mt^ response and the lifespan phenotypes upon depletion of NHR‐8 and SBP‐1. While in PHB‐deficient animals depletion of both TFs extend lifespan, in *sgk*‐*1* mutants depletion of NHR‐8 extends but depletion of SBP‐1 shortens lifespan. Only SBP‐1 was partially required for the longevity conferred by PHB depletion to *sgk*‐*1* mutants. Further supporting a role for sterol metabolism in mitochondrial stress, cholesterol supplementation alleviates the UPR^mt^ induction in PHB‐depleted animals, both, otherwise wild‐type worms and *sgk*‐*1* mutants. ATFS‐1 regulates hundreds of targets with a possible role in mitochondrial recovery (Nargund et al., 2015). Among them, ATFS‐1 induces enzymes of the mevalonate/isoprenoid/cholesterol synthesis pathway upon mitochondrial disfunction (Oks et al., 2018; Rauthan et al., 2013), which could contribute to the lifespan extension conferred by PHB depletion. Whether *sbp*‐*1* expression is directly or indirectly regulated by ATFS‐1 remains to be determined. Interestingly, ATFS‐1 regulates fat oxidation through the transcription factor HLH‐11, which was also a candidate of our screen (Littlejohn et al., 2020). Our data suggest that in *C. elegans*, SGK‐1 plays a key role in membrane lipid composition and sterol homeostasis and that organellar membrane contact sites could be modulated by TORC2/SGK‐1 to coordinate cellular stress responses. Whether induction of the UPR^mt^ is triggered by lipid bilayer stress or by the increased ROS or/and accumulation of unfolded proteins that can derive from altered membrane lipid homeostasis remains to be elucidated. Alternatively, the increased P*hsp*‐*6*::GFP levels observed in TORC2/SGK‐1 mutants could be due to the increased mitochondrial mass previously observed in *rict*‐*1* and *sgk*‐*1* mutants (Gatsi et al., 2014). The fact that the increased UPR^mt^ in TORC2/SGK‐1 deficient animals is ATFS‐1 independent supports this view.

We show that mitochondria‐associated ER membranes (MAM) are reduced in *sgk*‐*1* mutants, while in mammals, mTORC2 and SGK1 regulate mitochondrial function by maintaining MAM integrity (Betz et al., 2013). MAM have been shown to determine sites of mitochondrial fission (Friedman et al., 2011), therefore, the reduced MAM observed in *sgk*‐*1* mutants could be responsible for their increased mitochondrial size and interconnectivity. MAM mediate ER homeostasis and synthesis and maturation of key lipids including cholesterol, phospholipids and sphingolipids (Vance, 2014). Defects in MAM, and probably other ER membrane contacts, could explain the reduced lipid droplet and yolk/lipoprotein accumulation observed in *sgk*‐*1* mutants (Dowen et al., 2016; Wang et al., 2016; Yen et al., 2010) and the requirement of SGK‐1 in peripheral tissues for lipid storage (Mutlu et al., 2020). Moreover, they suggest that SGK1 could mediate the key role of mTORC2 in de novo lipid synthesis in mammals (Hagiwara et al., 2012; Martinez Calejman et al., 2020) and in cancer cells (Guri et al., 2017). Further supporting a role for SGK‐1 in ER homeostasis, the UPR^ER^ is induced in *sgk*‐*1* mutants (Zhu et al., 2015) and SGK1 regulates ER‐dependent gene expression (Toska et al., 2019). Strikingly, mitochondrial PHB dysfunction suppresses defects in lipogenesis and yolk/lipoprotein production. One interesting possibility is that lack of the PHB complex balances membrane lipid alterations in *sgk*‐*1* mutants. Although direct experimental evidence supporting this hypothesis is missing, the PHB complex affects membrane lipid composition and genetically interacts with proteins involved in ER‐mitochondria communication (Birner et al., 2003; Kornmann et al., 2009; Lourenco et al., 2015; Osman et al., 2009; Richter‐Dennerlein et al., 2014). Elucidating the molecular mechanisms behind this functional interaction deserves further investigation.

We observed accumulation of myelinated structures in *sgk*‐*1* mutants which could suggest a blockage in the last steps of the autophagy process, the degradation of the cargo, which might further implicate defective lysosomal function. PHB deficiency reduced the accumulation of myelinated forms in *sgk*‐*1* mutants. However, we failed to show alterations in the levels of the lysosomal protein LMP‐1 upon PHB depletion as well as differences at the level of autolysosomes in the double mutant. Instead, we detected a significant increase in Lysotracker staining upon PHB depletion in *sgk*‐*1* mutants, suggesting that PHB depletion might trigger signals than induce specific type of lysosomes or increase lysosomal acidity in *sgk*‐*1* mutants. Further experiments are required to analyse lysosomal composition and acidity in the different backgrounds, as well as the implication of lysosomes in the lifespan phenotypes. Nevertheless, our finding is promising in view of the reported role of lysosomes in regulating lifespan in a cross‐talk with mitochondrial function (Liu et al., 2020; Ramachandran et al., 2019; Tharyan et al., 2020).

Finally, we show that autophagy and the UPR^mt^/ATFS‐1, but not mitophagy, are required for the lifespan extension conferred by PHB depletion to *sgk*‐*1* mutants. Because ATFS‐1 is a positive regulator of autophagy (Nargund et al., 2015), inhibition of the UPR^mt^ could mimic inhibition of autophagy. However, inhibiting mitophagy does not cause additional stress (since autophagy is functional); instead, it might be beneficial to reduce mitochondrial clearance when mitochondria are severely damaged and biogenesis compromised. In fact, mitophagy is detrimental for both, PHB and *sgk*‐*1* single mutants. Recent data confirmed the beneficial effect of inhibiting mitophagy for *sgk*‐*1* development and reproduction (Aspernig et al., 2019) as well as lifespan (Zhou et al., 2019). Apart from the UPR^mt^, additional mechanisms could mediate autophagy induction. Upon lipid bilayer stress, lipid droplet formation and autophagy are induced through the IRE‐1/XBP‐1 axis (Koh et al., 2018). Also, HLH‐30 has been recently shown to mediate mitochondrial lifespan extension through activation of lysosomal biogenesis and autophagy (Liu et al., 2020).

In the future, it will be important to decipher the role of SGK‐1 at MAM at a molecular level and the mechanism by which PHB depletion suppresses *sgk*‐*1* defects. Given the implication of TORC2, PHB and autophagy in ageing and cancer, our findings may contribute to ameliorate the pathogenesis of ageing‐related disorders.

## 4 EXPERIMENTAL PROCEDURES

### 4.1 *C*. *elegans* strains and worm culture

We maintained nematodes at 20°C on nematode growth media (NGM) agar plates seeded with live *Escherichia coli* OP50‐1. A detailed description of the strains used, and the RNAi culture conditions are presented in the Supporting Information.

### 4.2 Imaging

On the day of imaging, worms were anaesthetized with 10 mM Levamisole (Sigma‐Aldrich) or with a mixture of 10 mM Levamisole and 5 mM NaN_3_ (Sigma‐Aldrich) for confocal imaging and mounted on 2% agarose pads. Details for each reporter quantified are described in Supporting Information.

### 4.3 Transmission electron microscopy

A detailed description of the TEM protocol and the quantification of different structures are presented in the Supporting Information.

### 4.4 Oxygen consumption

Worm oxygen consumption was measured using the Seahorse XFp Analyzer (Agilent). Worms at the young adult stage (30 per well) or day 6 adult worms (20 per well), were transferred into M9‐filled Seahorse plates. Values were normalized to protein content (Bicinchoninic acid assay, BCA, Thermo Fisher Scientific). A detailed description is presented in the Supporting Information.

### 4.5 Quantification of reactive oxygen species

Cytoplasmic ROS was quantified using 2,7‐dichlorofluorescin‐diacetate (H2‐DCFDA; Sigma‐Aldrich) as described (Artal‐Sanz & Tavernarakis, 2009b). Values were normalized to protein content (BCA). At least three independent assays were carried out, and the combined data were analysed by *t* test using GraphPad Prism software (version 5.0a).

### 4.6 Quantification of autophagy

In order to quantify autophagy flux, we used the reporter Ex[*Pnhx*‐*2*::mCherry::LGG‐1]. Animals were classified in 5 different expression categories (Figure S4a). Expression categories and Bafilomycine treatment are described in detail in Supplementary information. To quantify autophagosomes (AP) and autolysosomes (AL), we used the dual reporter P*lgg*‐*1*::mCherry::GFP::LGG‐1. Details are described in Supplementary information.

### 4.7 Lifespan analysis

Lifespans were performed at 20°C. Details and statistics (Tables S1 and S2) are described in Supporting Information.

### 4.8 RNAi screen

We performed an image‐based RNAi screen as previously described (Hernando‐Rodriguez et al., 2018). We tested 836 RNAi clones in duplicate and candidates were defined based on the adjusted *p* value and the fold change (FC; *p* < 0.001 and FC <0.6). Details are described in Supporting Information.

## CONFLICT OF INTEREST

The authors declare that they have no conflict of interest.

## AUTHOR CONTRIBUTIONS

P.dl.C.R., B.H.R., M.M.P.J. and M.A.‐S. designed the experiments. P.dl.C.R., B.H.R., M.M.P.J., M.J.R.‐P., M.D.M.‐B., A.P., R.G. and M.A.‐S. carried out experiments, analysed the data and interpreted results. B.H.R. and M.A.‐S. wrote the manuscript. All authors read, commented and approved the final manuscript.

## Supporting information

Fig S1

Fig S2

Fig S3

Fig S4

Fig S5

Table S1‐S2

## Data Availability

The data that support the findings of this study are available from the corresponding author upon reasonable request.
