# Supplementary figures and images for: Prohibitin depletion extends lifespan of a TORC2/SGK‐1 mutant through autophagy and the mitochondrial UPR
Source: Aging Cell. 2021 May 3;20(5):e13359. doi: 10.1111/acel.13359 (PMC8135086; doi:10.1111/acel.13359)

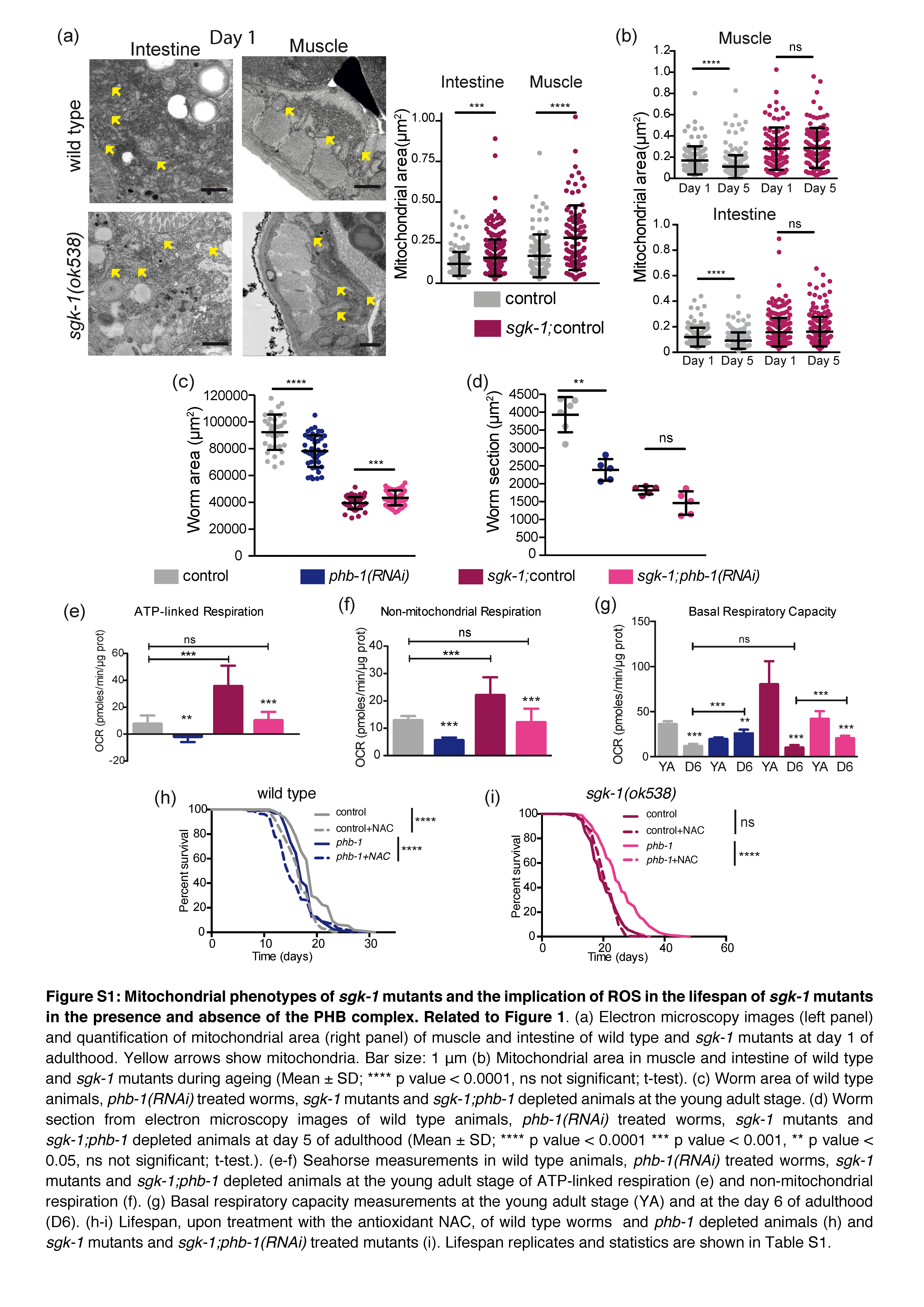

Supplement: Supplementary file 1 — Fig S1 [file ACEL-20-e13359-s005.tiff]

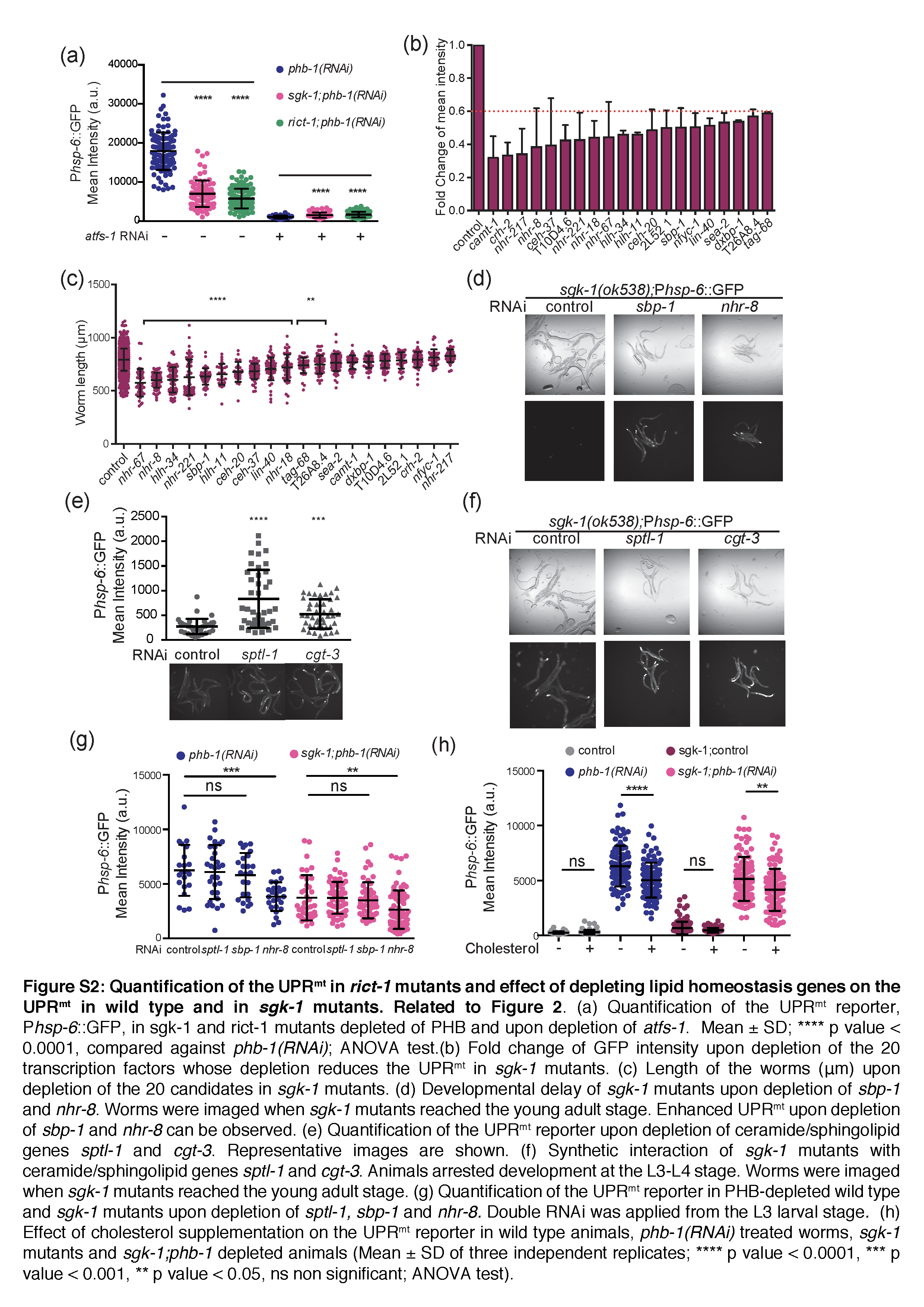

Supplement: Supplementary file 2 — Fig S2 [file ACEL-20-e13359-s001.tiff]

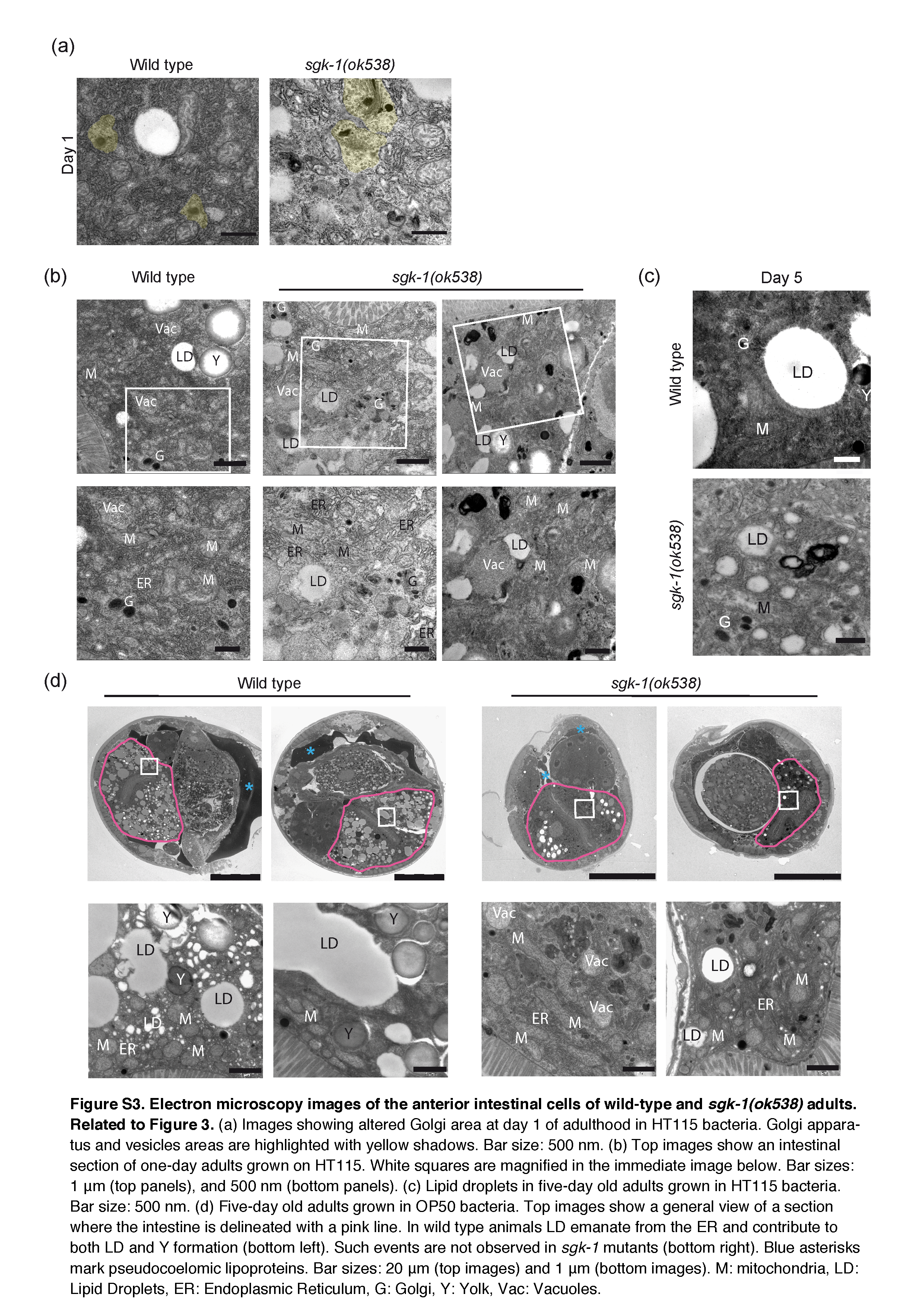

Supplement: Supplementary file 3 — Fig S3 [file ACEL-20-e13359-s006.tiff]

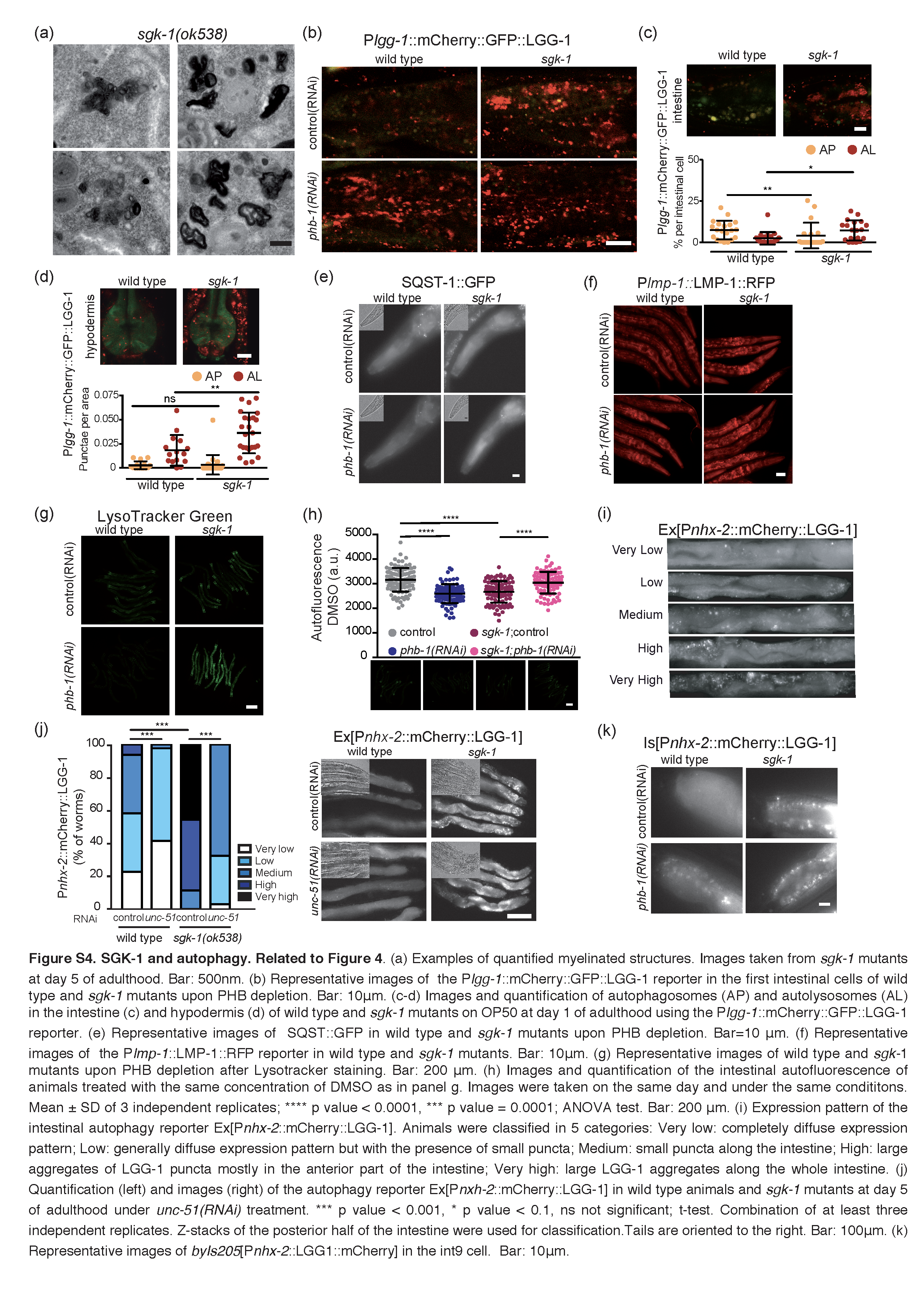

Supplement: Supplementary file 4 — Fig S4 [file ACEL-20-e13359-s002.tiff]

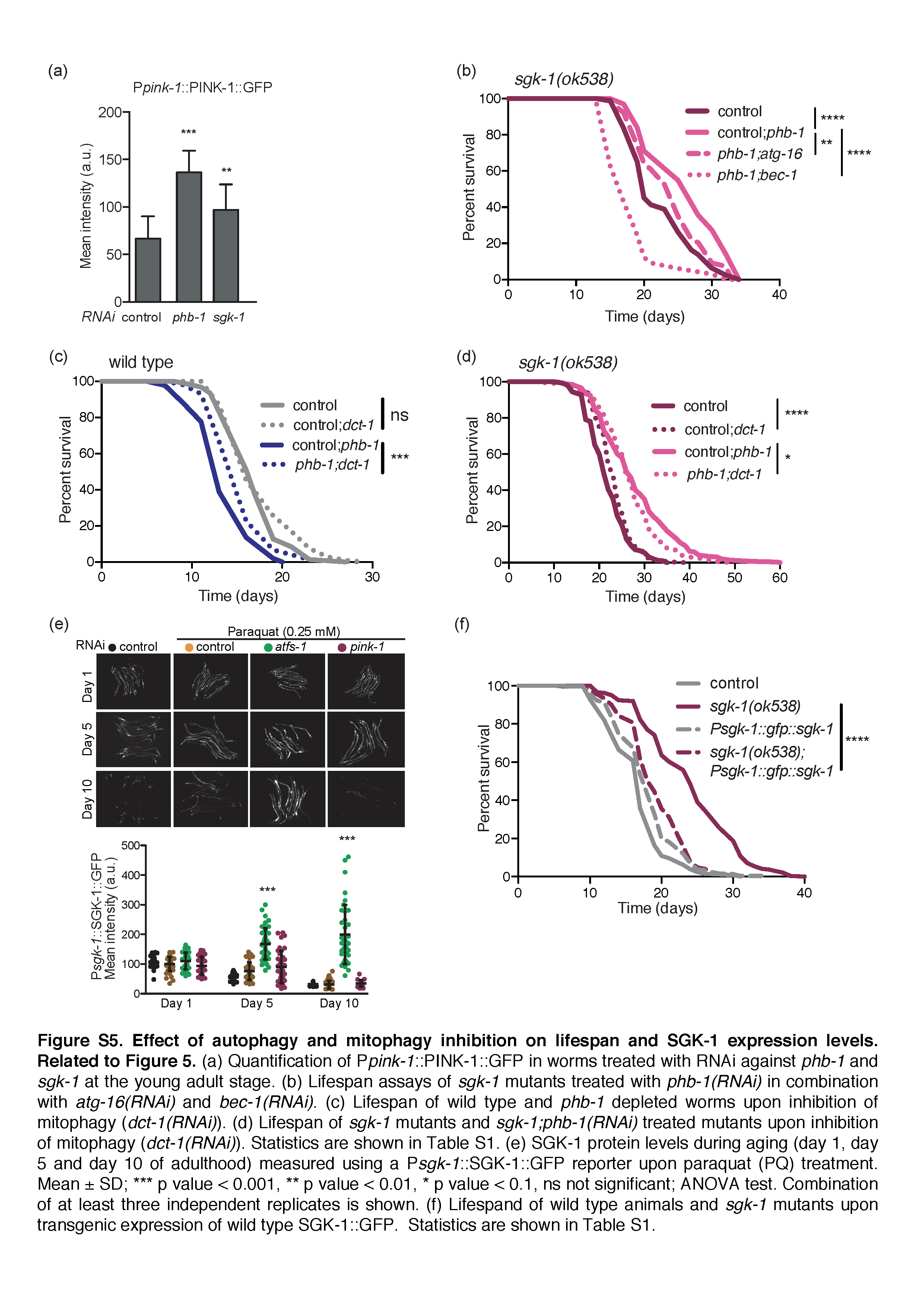

Supplement: Supplementary file 5 — Fig S5 [file ACEL-20-e13359-s003.tiff]
