# Supplementary material for: Prohibitin depletion extends lifespan of a TORC2/SGK‐1 mutant through autophagy and the mitochondrial UPR
Source: Aging Cell. 2021 May 3;20(5):e13359. doi: 10.1111/acel.13359 (PMC8135086; doi:10.1111/acel.13359)
Supplement: Supplementary file 6 — Table S1‐S2 [file ACEL-20-e13359-s004.pdf]

Supporting information

Prohibitin depletion extends lifespan of a TORC2/SGK-1 mutant by suppressing autophagy defects

Patricia de la Cruz Ruiz[1,2,*], Blanca Hernando-Rodríguez[1,2,*], Mercedes M. Pérez-Jiménez[1,2,*], María Jesús Rodríguez-Palero[1,2], Manuel D. Martínez-Bueno[1,2], Antoni Pla[1,2], Roxani Gatsi[1,2] and Marta Artal-Sanz[1,2 #]

[1]Andalusian Centre for Developmental Biology, Consejo Superior de Investigaciones Científicas/Junta de Andalucía/Universidad Pablo de Olavide, Seville, Spain

[2]Department of Molecular Biology and Biochemical Engineering, Universidad Pablo de Olavide, Seville, Spain

[#]Correspondence to: Marta Artal-Sanz. martsan@upo.es. Andalusian Centre for Developmental Biology. University Pablo de Olavide. Ctra. De Utrera km.1.

41013 Sevilla. Spain.

*Equal contribution

This file contains:

Supporting text: Detailed Experimental Procedures

5 Supplementary Figures

1 Supplementary Table

C. elegans strains

The *C. elegans* strains used in this study were: N2 (wild type); MRS486: *sgk-1(ok538)X* (3x outcrossed from BR4774, in total 11x outcrossed to N2 from the CGC strain VC345; MRS88: *daf-2(e1370)III;* MRS68: *daf-2(e1370)III*;*sgk-1(ok538)X;* MRS386: *byEx*[*Psgk-1::*SGK-1*::*GFP*+rol-6(su1006)* (1x outcrossed from BR2773 (Hertweck et al., 2004))*;* MRS38: *daf-2(e1370)III*;*byEx[*P*sgk-1::*SGK-1::GFP*+rol-6(su1006)];* VK1093: *vkEx1093[*P*nhx-2::*mCherry::LGG1]; MRS92: *sgk-1(ok538)X;vkEx1093[*P*nhx-2::*mCherry::LGG1]; MRS112: *byEx[*P*sgk-1::*SGK-1::GFP *+ rol-6(su1006)]; vkEx1093[*P*nhx-2::*mCherry::LGG1]; SJ4100: *zcIs13[*P*hsp-6::*GFP*]V;* MRS19: *sgk-1(ok538)X*;*zcIs13[*P*hsp-6::*GFP*]V;* MRS63: *rict-1(ft7)II*;*zcIs13[*P*hsp-6::*GFP*]V*; BR4006: *pink-1(tm1779)II;byEx655[*P*pink-1::*PINK-1::GFP *+* P*myo-2::*mCherry + herring sperm DNA]; MAH215: *sqIs11*[P*lgg-1*::mCherry::GFP::LGG-1+*rol-6(su1006)*]; MRS555: *sgk-1(ok538)X;sqIs11*[P*lgg-1*::mCherry::GFP::LGG-1+*rol-6(su1006)*]; MAH240: *sqIs17*[P*hlh-30*::HLH-30::GFP + *rol-6(su1006)*]; MRS558: *sgk-1(ok538)X;sqIs17*[P*hlh-30*::HLH-30::GFP + *rol-6(su1006)*]; MRS505: *sod-1(tm776)II* (2x outcrossed to N2); MRS516: *sod-1(tm776)II;sgk-1(ok538)X*; MRS504: *sod-2(gk257)I* (2x outcrossed to N2); MRS510: *sod-2(gk257)I;sgk-1(ok538)X*; MRS158:_*zcIs14*[*myo-3::*GFP]*;* MRS437: *sgk-1(ok538);zcIs14*[P*myo-3::*GFP]*;* MRS550: *unc-119(ed3)III;cdIs194[*P*lmp-1::lmp-1::*RFP*(S158T);unc-119(+)-ttx-3::gfp]*; MRS561: *sgk-1(ok538)X;unc-119(ed3)III;cdIs194[*P*lmp-1::lmp-1::*RFP*(S158T);unc-119(+)-ttx-3::gfp];* VZ827: *bpIs151[Psqst-1::sqst-1::gfp;unc-76]IV*; MRS571: *sgk-1(ok538)X;bpIs151[Psqst-1::sqst-1::*gfp*;unc-76]IV;* BR7019: *byIs205* [*Pnhx-2::mCherry::lgg-1*]; BR7042: *sgk-1(ok538)X; byIs205* [*Pnhx-2::mCherry::lgg-1*].

Strains generated by injection of a plasmid carrying wild type *sgk-1* cDNA (pBY3667)(a gift from Prof. Ralf Baumeister): MRS583: *sevEx8*[*Psgk-1::pEGFP-C1::sgk-1;*pRF4*];* MRS581: *sgk-1(ok538);sevEx8*[*Psgk-1::pEGFP-C1::sgk-1;*pRF4*];* MRS584: *sgk-1(ok538) sevEx7[Plmn-1::gfp::his-58;pRF4];* MRS586: *sevEx7[Plmn-1::gfp::his-58;pRF4].*

RNAi assays on plates

Worms were placed on NGM plates, supplemented with 25 μg/ml carbenicillin (Sigma-Aldrich) and 10 μg/ml nystatin (Sigma-Aldrich), seeded with HT115 (DE3) *Escherichia coli* bacteria transformed with empty vector (control) or the target gene RNAi construct. Each bacterial strain was inoculated in LB (ampicillin (100 μg/ml) (Sigma-Aldrich) and tetracycline (15 μg/ml) (Sigma-Aldrich) from an overnight pre-inoculum (1:10 dilution) and grown at 37°C for 3 hours, until an OD_{600} of around 1.5. We added IPTG (1 mM final concentration) (Sigma-Aldrich) to the bacterial culture and harvested cells by centrifugation (8 min at 6000×*g,* 4°C) after 2 hours at 37°C. Following, we washed the pellets with S Basal and harvested them again. Finally, we re-suspended bacterial pellets to a final concentration of 30 g/l in complete S Medium. Bacterial stocks were kept at 4°C up to 4 days before being used. For double RNAi treatments, bacterial stocks were mixed in a proportion of 1:1 before seeding the plates. A semi-synchronous embryo population was grown on plates seeded with the appropriate RNAi bacterial clone at 20°C until the desired stage (young adult, day 1, day 5 or day 10 of adulthood). During egg-laying, we transferred worms every day and every 2 days thereafter. To pharmacologically induce a strong mitochondrial stress, we transferred L3 to fresh RNAi plates containing 0.25 mM Paraquat (Sigma-Aldrich).

Transcription Factor RNAi screen

We dispensed 60 synchronized *sgk-1;Phsp-6::gfp* L1 larvae per well in 96 well plates using a microplate dispenser (EL406 washer dispenser, BioTek) and added 75 µl of bacterial cultures from a Transcription-Factor RNAi sub-library. We used an empty feeding vector, pL4440, as negative control (control RNAi) and *phb-1* RNAi as positive control. Two independent replicates were carried out. We incubated worms at 20ºC with shaking (120 rpm - New Brunswick™ Innova® 44/44R) until they reached the young adult stage. Worms were immobilised with levamisole and plates were washed with water to eliminate bacteria before imaging. We acquired pictures of each well using the IN Cell Analyzer 2000 (GE Healthcare) and performed the image analysis with a user-defined protocol which was developed on the Developer Toolbox software (GE Healthcare).

For the statistical analysis, outliers were removed by excluding the 5th and the 95th percentile of the distribution. Wells with less than 10 worms were removed from the analysis. A quality assay

was performed in the control wells: only control wells with mean GFP intensity between 200 and 500 a.u. and a coefficient of variation < 0.5 were accepted. If less than 2 control wells remained accepted, the plate was discarded and repeated. In order to make data from different plates comparable, data are normalized by dividing the GFP value of each worm by the mean of the GFP of the four negative control wells. Finally, statistics are assessed by running an ANOVA test followed by a Dunnett's test. Candidates are defined based on the *p* value and the fold change (FC) (p value $< 10^{-4}$ and FC < 0.6).

Cholesterol supplementation

Nematode were grown from eggs in NGM agar plates, seeded with RNAi bacteria, and supplemented with cholesterol at 25 mg/ml (high cholesterol) or 5 mg/ml (and equivalent amount of vehicle).

Imaging

P*pink-1::*PINK-1::GFP, Ex[P*nhx-2::*mCherry::LGG1], *Is*[*Pnhx-2*::mCherry::LGG-1], *bpIs[Psqst-1*::*sqst-1*::GFP*]* and P*sgk-1::*SGK-1::GFP reporters were observed using the AxioCam MRm camera on a Zeiss ApoTome Microscope; P*lgg-1*::mCherry::GFP::LGG-1 reporter was imaged with a confocal Nikon A1R equipped with a Plan Apo VC 60x/1.4 objective and P*hsp-6::*GFP. P*lmp-1*::*lmp-1*::RFP(S158T)*,* Lysotracker stained animals and P*hlh-30*::HLH-30::GFP animals were visualized with a Leica M205 Stereoscope equipped with a Plan Apo 5.0x/0.50 LWD objective and a ORCA-Flash4.0 LT Hamamatsu digital camera. Image analysis was performed using the ImageJ software. Emission intensity was measured on greyscale images with a pixel depth of 16 bits. At least two independent assays were carried out and the combined data was analysed using the GraphPad Prism software (version 5.0a).

Imaging and quantification of mitochondrial morphology

zcIs14 [P*myo-3*::GFP(mit)] and *sgk-1(ok538);zcIs14*[P*myo-3*::GFP(mit)] reporters were mounted on 2% agarose pads using 40 mM levamisol as anaesthetic. Mitochondria in the body wall muscle

were imaged, except around the vulva and pharyngeal regions, using a confocal Nikon A1R equipped with a Plan Apo VC 60x/2 objective. We used ImageJ to remove background, to apply median filter and to convert to a binary image by auto-thresholding. Images (whole cells) were segmented and area and perimeter per segment were calculated. Mean area/perimeter ratio was calculated to represent the mitochondrial interconnectivity. Data was analyzed using the GraphPad Prism software (version 6.0c).

Quantification of HLH-30

Animals were mounted at day 1 of adulthood and anaesthetised with 10mM levamisole Given the strong effect of the anaesthetics in triggering HLH.30 nuclear localization, we imaged all the worms within 2 minutes from the time they were removed from food. The two first intestinal cells of P*hlh-30*::HLH-30::GFP animals were analysed and classified in 3 depending on the nuclear accumulation of HLH-30 GFP signal: Cytoplasmic (very low nuclear signal), Intermediate (middle level of nuclear signal) and Nuclear (high level of nuclear signal). At least 4 independent replicas (n= 114 and n= 119 for wild type and PHB-depleted animals, respectively, and n= 175 and n= 189 for *sgk-1* and *sgk-1* PHB-depleted animals, respectively).

Quantification of *lmp-1*:: RFP

Animals were mounted at the young adult stage and anaesthetised with 10mM levamisole. We analysed the first two intestinal cells of wild type and *sgk-1* animals under control conditions and upon *phb-1* depletion. At least 4 independent replicas between 20-30 worms per condition (n= 73 and n=90 for wild type and PHB-depleted worms, respectively, and n=70 and n=78 for *sgk-1* and *sgk-1* PHB-depleted animals, respectively).

Lysotracker staining

Lysotracker Green DND-26, 1 mM solution in DMSO, (Invitrogen) was diluted in M9 Buffer as working solution 100mM. Young adults were placed onto RNAi-bacteria seeded plates containing 1 mM of Lysotracker. RNAi-bacteria seeded plates containing the equivalent amount of vehicle were used as control. After 16 hours worms were transfer to fresh RNAi-bacteria seeded plate

without Lysotracker to clear intestinal lumen from excess dye. Image analysis was performed using the ImageJ software. Lysotracker and DMSO-only containing plates were imaged under the same conditions. Three independent assays were carried out and the combined data was analysed using the GraphPad Prism software (version 5.0a).

Transmission electron microscopy

Adult worms were immersed in 0.8% glutaraldehyde + 0.8% osmium tetroxide in 0.1 M sodium cacodylate buffer, pH7.4 and cut with a scalp at the posterior end of the intestine. We kept samples for 1 hour on ice under dark conditions to allow complete penetration of the fixing solution. We washed worms three times with 0.1 M sodium cacodylate buffer and fixed them over night at 4°C in 2% osmium tetroxide in 0.1 M sodium cacodylate buffer; the entire procedure was performed on ice. On the next day, we washed again three times with 0.1 M sodium cacodylate buffer on ice and embedded the worms in small cubes of 1% agarose. Then, samples where dehydrate; incubated in 50%, 70% and 90% ethanol for 10 minutes each, followed by 3 washes of 10 minutes each in 100% ethanol, at room temperature. Next, samples where incubated in 50:50 ethanol/propylene oxide solution for 15 minutes followed by 2 incubations of 15 minutes each in 100% propylene oxide. Samples were infiltrated on a rotator or with an embedding machine for 2 hours in a 3:1 ratio of propylene oxide to resin and next for 2 hours in a 1:3 ratio of propylene oxide to resin. Next, samples where incubated overnight in 100% resin and the next day changed to fresh 100% resin for 4 hours. Finally, samples were arranged in a flat embedding mold and cured at 60°C for 2 days. Worms were cut at the level of the first intestinal cells, immediately before and after the gonad turn, specifically at 175 μm, 185 μm, 250 μm and 350 μm from the mouth.

Quantification of transmission electron microscopy structures.

For mitochondrial area quantification, 2 and 4 different animals were analyzed for D1 and D5 adulthood respectively; $n \geq 92$ mitochondria per background were measured. For Golgi area quantification, 2 to 3 animals were analyzed, $n \geq 20$ mitochondria per background. To determine

ER-mitochondria contact size, a maximal distance of 50 nm between organelles was considered as a contact; n between 22 and 107 mitochondria per background, from 2 to 3 animals. ER-mitochondria contact site lengths were normalized to mitochondrial perimeters. To quantify myelinated forms we analysed 12 areas of 56 μm^2 per condition and counted complex myelinated autophagic structures and myelinated autolysosomes (Figure S4d). Data was analyzed using the GraphPad Prism software (version 5.0a).

Oxygen consumption

OCR was measured 8 times in basal conditions as well as after each injection. Working solutions were diluted in M9 at the final concentrations: FCCP (Sigma-Aldrich) 250 µM, oligomycin (Sigma-Aldrich) 250 µM and NaN_3 (Sigma-Aldrich) 400 mM. Basal OCR corresponds to the average of 8 measurements under basal conditions. After drug addition, the values derived from the average of the last 4 measurements. At least three independent assays were carried out and the combined data was analysed by *t*-test using GraphPad Prism software (version 5.0a).

Quantification of Autophagy

Ex[*Pnhx-2*::mCherry::LGG-1] expression categories: Very low: completely diffused expression pattern; Low: generally diffuse expression pattern with the presence of small puncta; Medium: puncta along the intestine; High: aggregation of LGG-1 puncta mostly in the posterior part of the intestine; Very high: large LGG-1 aggregates along the whole intestine. To avoid bias in the classification process, different researchers performed blind scoring on the same animal images and disputes were resolved after discussion. For Bafilomycine A1 (BafA; Sigma-Aldrich) treatment, day 5 adult worms were washed from plates with M9 buffer, washed three times with M9 and incubated 6 hours in M9 with BafA (25 *µ*M final concentration) or DMSO (1.5%) at 20°C with agitation. At least 3 independent assays were carried out and the combined data was analysed by t-test using GraphPad Prism software (version 5.0a).

byIs205 [*Pnhx-2::mCherry::lgg-1*] and *bpIs151[Psqst-1::sqst-1::GFP]* expression pattern was analysed at Day 1. Worms were anesthetized in NaN_3 (25 to 50 mM). *Is*[*Pnhx-2::mCherry::lgg-1*]

worms were imaged with a 63x objetive. mCherry punctae within one int9 cell, per one focal plane, were quantified. *[Psqst-1::sqst-1::gfp]* worms were imaged with a 40x objetive. Z-stacks of the head region were acquired and maximum intensity projection was applied. GFP loci within the head region were quantified. At least 3 independent assays were carried out and the combined data was analysed by t-test using GraphPad Prism software (version 5.0a)

Quantification of autophagosomes and autolysosomes

To quantify autophagosomes (AP) and autolysosomes (AL) we used the dual reporter *sqIs11*[P*lgg-1*::mCherry::GFP::LGG-1]. We quantified the number of punctae of AL/AP (red or yellow punctae respectively) of the two first intestinal cells in wild type and *sgk-1* animals under control RNAi (n= 25, 31 respectively) and *phb-1* RNAi (n= 31, 26 respectively). Three independent replicas were analysed. In OP50, AP and AL were analysed in the intestine and hypodermis (head region) of wild type and *sgk-1* mutants. Two independent replicas, n= 7 for wild type and n=14 for *sgk-1(ok538).*

For the quantification we used ImageJ software, selecting a specific stack and a defined area occupied by the two first intestinal cells for each worm, after processing with a gaussian filter (sigma radius: 1) and selecting a fixed threshold for all images. Finally, we performed an automatic particle counting using "analize particle" plugin with a previous watershed segmentation.

Lifespan Analysis

Synchronized eggs were obtained by hypochlorite treatment of adult hermaphrodites and placed on NGM plates seeded with HT115(DE3) *E. coli* bacteria, carrying appropriate RNAi plasmid constructs. RNAi was applied from the L1 larval stage, except for *sgk-1* mutants on *sptl-1*, *nhr-8* or *sbp-1* RNAi, where RNAi was applied from the late-L3 larval stage. Adults were transferred to fresh plates every day during the reproductive period and afterwards on alternate days. Worms were scored as dead when they no longer responded to touch. Exploded animals or those exhibiting bagging were censored. For N-Acetyl-L-Cysteine (NAC) (Sigma-Aldrich) treatments we added NAC to a final concentration of 8 mM to NGM plates. We used the GraphPad Prism software (version 5.0a) to plot survival curves and to determine significant differences in lifespans (log-rank (Mantel-

Cox) test). See Table S1 for lifespan statistics.

Table S1: Lifespan data

				P value	
Strain	Median ± SEM (days)	$ Max ± SEM (days)	# deaths/total	*vs ctrl* RNAi	*vs phb-1* RNAi/NAC-
wild type,*ctrl* RNAi	16 ± 1	19 ± 2	160 / 320 (2)		
wild type,*ctrl_phb-1* RNAi	14 ± 0	16.5 ± 0.5	101 / 320 (2)	< 0.0001 (****)	
wild type,*ctrl_unc-51* RNAi	14 ± 0	16.5 ± 0.5	75 / 320 (2)	< 0.0001 (****)	
wild type,*phb-1_unc-51* RNAi	13 ± 0	15.5 ± 1.5	64 / 320 (2)		0.2935 (ns)
sgk-1(ok538),ctrl RNAi	21.5 ± 1.5	27 ± 0	133 / 240 (2)		
sgk-1(ok538),ctrl_phb-1 RNAi	23.5 ± 0.5	30 ± 2	143 / 240 (2)	0.0013 (**)	
sgk-1(ok538),ctrl_unc-51 RNAi	22.5 ± 1.5	25.5 ± 0.5	95 / 240 (2)	0.1403 (ns)	
sgk-1(ok538),phb-1_unc-51 RNAi	19.5 ± 0.5	23.5 ± 0.5	149 / 240 (2)		< 0.0001 (****)
wild type,*ctrl* RNAi	18 ± 1	23.33 ± 1.20	336 / 489 (3)		
wild type,*ctrl_phb-1* RNAi	16 ± 2	19.5 ± 2.5	186 / 340 (2)	< 0.0001 (****)	
wild type,*ctrl_atfs-1* RNAi	18.33 ± 0.88	23.67 ± 0.88	350 / 490 (3)	0.5436 (ns)	
wild type,*phb-1_atfs-1* RNAi	16 ± 2	20 ± 2	259 / 340 (2)		0.5462 (ns)
sgk-1(ok538),ctrl RNAi	21.5 ± 1.5	27 ± 0	252 / 432 (2)		
sgk-1(ok538),ctrl_phb-1 RNAi	23.5 ± 0.5	30 ± 2	274 / 440 (2)	< 0.0001 (****)	
sgk-1(ok538),ctrl_atfs-1 RNAi	22 ± 1	26 ± 0	248 / 440 (2)	0.5285 (ns)	
sgk-1(ok538),phb-1_atfs-1 RNAi	18 ± 1	22.5 ± 0.5	300 / 439 (2)		< 0.0001 (****)
wild type,*ctrl* RNAi	17 ± 0	21 ± 0	181 / 275 (2)		
wild type,*ctrl_phb-1* RNAi	13.5 ± 0.5	17 ± 0	118 / 262 (2)	< 0.0001 (****)	
wild type,*ctrl_pink-1* RNAi	16.5 ± 0.5	21.5 ± 0.5	218 / 285 (2)	0.3655 (ns)	
wild type,*phb-1_pink-1* RNAi	16 ± 0	20 ± 1	115 / 236 (2)		< 0.0001 (****)
sgk-1(ok538),ctrl RNAi	21.8 ± 0.97	28.4 ± 1.12	533 / 1040 (5)		
sgk-1(ok538),ctrl_phb-1 RNAi	26.4 ± 1.29	34.2 ± 2.06	543 / 1029 (5)	< 0.0001 (****)	
sgk-1(ok538),ctrl_pink-1 RNAi	24 ± 0.45	29.2 ± 0.86	480 / 951 (5)	0.0001 (***)	
sgk-1(ok538),phb-1_pink-1 RNAi	27 ± 1.41	32.75 ± 2.06	450 / 840 (4)		0.5699 (ns)
wild type,*ctrl* RNAi	17	21	75 / 115 (1)		
wild type,*ctrl_phb-1* RNAi	13	17	56 / 102 (1)	< 0.0001 (****)	
wild type,*ctrl_dct-1* RNAi	16	23	107 / 125 (1)	0.1355 (ns)	
wild type,*phb-1_dct-1* RNAi	16	19	80 / 123 (1)		< 0.0004 (***)
sgk-1(ok538),ctrl RNAi	21.75 ± 1.25	27.75 ± 1.55	482 / 618 (4)		
sgk-1(ok538),ctrl_phb-1 RNAi	27.33 ± 1.76	38 ± 1	344 / 466 (3)	< 0.0001 (****)	
sgk-1(ok538),ctrl_dct-1 RNAi	23.25 ± 0.75	28.75 ± 1.03	504 / 617 (4)	< 0.0001 (****)	
sgk-1(ok538),phb-1_dct-1 RNAi	26.33 ± 0.33	35 ± 1.73	310 / 469 (3)		0.0479 (*)
wild type,*ctrl* RNAi_NAC-	18.66 ± 0.33	22.67 ± 0.88	218 / 426 (3)		
wild type,*ctrl* RNAi_NAC+	16.33 ± 0.67	19.33 ± 0.33	199 / 355 (3)		< 0.0001 (****)
wild type,*ctrl_phb-1* RNAi_NAC-	17.25 ± 0.63	20.5 ± 0.96	298 / 593 (4)	< 0.0001 (****)	
wild type,*ctrl_phb-1* RNAi_NAC+	14.5 ± 1.04	19.75 ± 2.21	357 / 583 (4)		< 0.0001 (****)
sgk-1(ok538),ctrl RNAi_NAC-	20.33 ± 2.4	27.33 ± 2.40	329 / 426 (3)		
sgk-1(ok538),ctrl RNAi_NAC+	20.66 ± 1.2	25 ± 1	357 / 426 (3)		0.0354 (ns)
sgk-1(ok538),ctrl_phb-1 RNAi_NAC-	22.5 ± 1.5	33 ± 2	307 / 426 (2)	< 0.0001 (****)	
sgk-1(ok538),ctrl_phb-1 RNAi_NAC+	20.5 ± 0.5	27 ± 1	222 / 276 (2)		< 0.0001 (****)
sgk-1(ok538), ctrl RNAi	20	30	82 / 308 (1)		
sgk-1(ok538), phb-1 RNAi	27	34	62 / 289 (1)	< 0.0001 (****)	
sgk-1(ok538),phb-1_atg-16 RNAi	25	30	58 / 280 (1)		0.0036 (**)
sgk-1(ok538),phb-1_bec-1 RNAi	17	21	34 / 133 (1)		< 0.0001 (****)
wild type,*ctrl* RNAi	19 ± 0	28 ± 0	287 / 360 (2)		
sod-1(tm776) ,ctrl RNAi	20 ± 1	28 ± 0	309 / 360 (2)	0.5597 (ns)	
sod-2(gk257) ,ctrl RNAi	23.5 ± 1.5	33 ± 1	319 / 360 (2)	< 0.0001 (****)	
sgk-1(ok538), ctrl RNAi	30.5 ± 1.5	37.5 ± 1.5	231 / 360 (2)		
sgk-1(ok538);sod-1(tm776) ,ctrl RNAi	27 ± 0	33 ± 1	265 / 360 (2)	< 0.0001 (****)	
sgk-1(ok538);sod-2(gk257) ,ctrl RNAi	32 ± 0	41 ± 0	251 / 360 (2)	< 0.0001 (****)	

wild type,*ctrl* RNAi	15.67 ± 0.89	19 ± 1.15	227 / 361 (3)		
wild type,*phb-1* RNAi	15 ± 1	20.33 ± 1.86	240 / 380 (3)	0.1890 (ns)	
sod-1(tm776),phb-1 RNAi	15 ± 1	19.66 ± 2.33	221 / 382 (3)		0.8978 (ns)
sod-2(gk257),phb-1 RNAi	15 ± 1	21.33 ± 1.86	260 / 380 (3)		0.2145 (ns)
sgk-1(ok538), ctrl RNAi	21 ± 2.52	25 ± 3	320 / 390 (3)		
sgk-1(ok538), phb-1 RNAi	26 ± 3.79	33 ± 3.79	220 / 380 (3)	< 0.0001 (****)	
sgk-1(ok538);sod-1(tm776) ,phb-1 RNAi	25.33 ± 2.03	36 ± 1.53	111 / 391 (3)		0.2174 (ns)
sgk-1(ok538);sod-2(gk257),phb-1 RNAi	22.33 ± 1.45	32.33 ± 3.33	255 / 400 (3)		0.0448 (*)
wild type,*ctrl* RNAi	16.67 ± 1.77	20 ± 2	270 / 360 (3)		
wild type,*ctrl_phb-1* RNAi	14.33 ± 1.33	17.66 ± 1.20	241 / 360 (3)	< 0.0001 (****)	
wild type,*ctrl_nhr-8* RNAi	15 ± 1	20 ± 2	269 / 360 (3)	0.0563 (ns)/0.0174(*)&	
wild type,*phb-1_nhr-8* RNAi	15.33 ± 2.4	19 ± 2.52	251 / 364 (3)		0.0004 (***)
sgk-1(ok538),ctrl RNAi	19.33 ± 1.86	24 ± 3	263 / 330 (3)		
sgk-1(ok538),ctrl_phb-1 RNAi	25.67 ± 3.28	34 ± 2.52	241 / 350 (3)	< 0.0001 (****)	
sgk-1(ok538),ctrl_nhr-8 RNAi	20 ± 1	32.67 ± 2.96	302 / 347 (3)	0.0198 (*)	
sgk-1(ok538),phb-1_nhr-8 RNAi	24.67 ± 2.03	36.33 ± 1.33	233 / 360 (3)		0.7669 (ns)
wild type,*ctrl* RNAi	18 ± 2	22.5 ± 1.5	195 / 241 (2)		
wild type,*ctrl_phb-1* RNAi	15.5 ± 1.5	19 ± 1	153 / 241 (2)	< 0.0001 (****)	
wild type,*ctrl_sbp-1* RNAi	17.5 ± 2.5	19 ± 3	164 / 240 (2)	< 0.0001 (****)	
wild type,*phb-1_sbp-1* RNAi	15.5 ± 1.5	19 ± 3	160 / 240 (2)		0.0209 (*)
wild type,*ctrl_sptl-1* RNAi	19.5 ± 2.5	24.5 ± 2.5	209 / 240 (2)	< 0.0001 (****)	
wild type,*phb-1_sptl-1* RNAi	15.5 ± 1.5	20 ± 4	166 / 247 (2)		0.1003 (ns)
sgk-1(ok538),ctrl RNAi	23 ± 0	29 ± 1	217 / 260 (2)		
sgk-1(ok538),ctrl_phb-1 RNAi	30 ± 2	40.5 ± 1.5	183 / 260 (2)	< 0.0001 (****)	
sgk-1(ok538),ctrl_sbp-1 RNAi	19 ± 2	21 ± 2	184 / 260 (2)	< 0.0001 (****)	
sgk-1(ok538),phb-1_sbp-1 RNAi	21 ± 0	27 ± 1	174 / 260 (2)		< 0.0001 (****)
sgk-1(ok538),ctrl_sptl-1 RNAi	25.5 ± 0.5	32 ± 0	190 / 260 (2)	< 0.0001 (****)	
sgk-1(ok538),phb-1_sptl-1 RNAi	29.5 ± 0.5	38 ± 1	153 / 263 (2)		0.5055 (ns)

All ageing experiments were performed on plates seeded with HT115(DE3) *E. coli*

P values were calculated using the log-rank test, except & (Gehan-Breslow-Wilcoxon test)

$ day where more than 90% of population is dead.

Confirmed death events divided by the total number of animals included the assay.

Total equals the number of animals that died plus the number of animals that were censored.

The number of independent lifespan assays for each strain is shown in parentheses.

Table S2: Lifespan rescue data

				P value	
Strain	Median ± SEM (days)	$ Max ± SEM (days)	# deaths/total	*vs* control	*vs sgk-1(ok538); Psgk-1::gfp::sgk-1*
control	17 ± 0	24 ± 0	262 / 360 (2)		< 0,0001(****)
sgk-1(ok538)	23 ± 2	33 ± 1	246 / 360 (2)	< 0.0001 (****)	< 0.0001 (****)
P*sgk-1::gfp::sgk-1*	18 ± 0	26 ± 1	293 / 360 (2)	< 0.0001 (****)	0,005(**)
sgk-1(ok538);P*sgk-1::gfp::sgk-1*	19 ± 1	25,5 ± 0.5	305 / 360 (2)	< 0.0001 (****)	

Ageing experiments were performed on plates seeded with HT115(DE3) *E. coli*

P values were calculated using the log-rank test

$ day where more than 90% of population is dead.

Confirmed death events divided by the total number of animals included the assay.

Total equals the number of animals that died plus the number of animals that were censored.

The number of independent lifespan assays for each strain is shown in parentheses.
